# Yip1A, a Novel Host Factor for the Activation of the IRE1 Pathway of the Unfolded Protein Response during *Brucella* Infection

**DOI:** 10.1371/journal.ppat.1004747

**Published:** 2015-03-05

**Authors:** Yuki Taguchi, Koichi Imaoka, Michiyo Kataoka, Akihiko Uda, Daiki Nakatsu, Sakuya Horii-Okazaki, Rina Kunishige, Fumi Kano, Masayuki Murata

**Affiliations:** 1 Department of Life Sciences, Graduate School of Arts and Sciences, The University of Tokyo, Meguro, Tokyo, Japan; 2 Department of Veterinary Science, National Institute of Infectious Diseases, Shinjuku, Tokyo, Japan; 3 Department of Pathology, National Institute of Infectious Diseases, Shinjuku, Tokyo, Japan; 4 PRESTO, Japan Science and Technology Agent, Kawaguchi, Saitama, Japan; University of California, Davis, UNITED STATES

## Abstract

*Brucella* species replicate within host cells in the form of endoplasmic reticulum (ER)-derived vacuoles. The mechanisms by which the bacteria are sequestered into such vacuoles and obtain a continuous membrane supply for their replication remain to be elucidated. In the present study, we provided several lines of evidence that demonstrate the mechanism by which *B*. *abortus* acquires the ER-derived membrane. First, during *Brucella* infection, the IRE1 pathway, but not the PERK and ATF6 pathways, of the unfolded protein response (UPR) was activated in a time-dependent manner, and the COPII vesicle components Sar1, Sec23, and Sec24D were upregulated. Second, a marked accretion of ER-derived vacuoles was observed around replicating bacteria using fluorescent microscopy and electron microscopy. Third, we identified a novel host factor, Yip1A, for the activation of the IRE1 pathway in response to both tunicamycin treatment and infection with *B*. *abortus*. We found that Yip1A is responsible for the phosphorylation of IRE1 through high-order assembly of Ire1 molecules at ER exit sites (ERES) under the UPR conditions. In Yip1A-knockdown cells, *B*. *abortus* failed to generate the ER-derived vacuoles, and remained in endosomal/lysosomal compartments. These results indicate that the activation of the IRE1 pathway and the subsequent formation of ER-derived vacuoles are critical for *B*. *abortus* to establish a safe replication niche, and that Yip1A is indispensable for these processes. Furthermore, we showed that the autophagy-related proteins Atg9 and WIPI1, but not DFCP1, were required for the biogenesis of the ER-derived membrane compartments. 　On the basis of our findings, we propose a model for intracellular *Brucella* replication that exploits the host UPR and ER-derived vacuole formation machineries, both of which depend on Yip1A-mediated IRE1 activation.

## Author Summary

The genus *Brucella* is a serious intracellular pathogen that causes brucellosis in a wide range of animals including humans. Infection with *Brucella* spp. results in a significant economic and health burden due to its high infectivity, chronic nature, and difficulties in vaccine production. Better understanding of the host-pathogen interplay that supports *Brucella* replication is essential for the development of effective treatments for brucellosis. The unfolded protein response (UPR) has been implicated in the pathogenesis of several viral and bacterial infections. These pathogens modulate individual pathways of the UPR to enable their replication in host cells. Autophagy has also been linked to the survival of several intracellular pathogens. They subvert autophagic machineries of host cells to establish their safe replication niche. In the present study, we show that the activation of the IRE1 pathway of the UPR and the subsequent formation of ER-derived vacuoles are crucial for intracellular survival of *B*. *abortus*. In addition, we identified a novel host factor Yip1A that is responsible for these processes. Characterization of the function of Yip1A will provide new insights into the molecular mechanisms by which *Brucella* spp. replicates in host cells.

## Introduction

The genus *Brucella* is a gram-negative facultative pathogen that causes a zoonotic disease known as brucellosis in a wide range of animals, including cows, goats, sheep, dogs and pigs, as well as humans [1]. *Brucella* infection causes abortion and sterility in animals, and debilitating disorders in humans. The high levels of infectivity of the pathogen, chronic nature of the infection, and difficulties in vaccine production cause a significant economic and health burden. Better understanding of the host–pathogen interplay that supports *Brucella* replication is essential for the development of effective treatments for brucellosis.

*Brucella* spp. can replicate in both phagocytic and non-phagocytic cells. Once within host cells, it resides in a membrane-bound compartment called the *Brucella*-containing vacuole (BCV). BCVs undergo a series of interactions with vesicular trafficking pathways in host cells. They first interact with early and late endosomes, and with lysosomes in a limited way [2], and then are targeted to the ER where they interact with ER exit sites (ERES), which are subdomains of the ER where dynamic membrane fission events occur [3]. The interaction of BCVs with ERES leads to fusogenic events between the BCVs and ER membranes, which thereby generate ER-derived replicative BCVs [3–6]. Celli et al. [3] suggested that functional ERES and specific interaction with COPII compartments at ERES are required for the biogenesis of replicative BCVs.

The unfolded protein response (UPR) has been implicated in the pathogenesis of several viral and bacterial infections, such as those of influenza A virus [7], hepatitis C virus [8], Japanese encephalitis virus [9], *Mycobacterium tuberculosis* [10], and group A *Streptococcus* [11]. These pathogens modulate individual pathways of the UPR in distinct ways to enable their replication in host cells. In mammalian cells, the UPR is composed of three pathways that are initiated by distinct ER sensors: inositol-requiring enzyme 1 (IRE1), protein kinase RNA (PKR)-like ER kinase (PERK), and activating transcription factor-6 (ATF6) [12]. These sensors are usually held in an inactive state by immunoglobulin binding protein (Bip). Under conditions of ER stress, Bip is released from the ER sensors, which allows activation of the UPR. Both IRE1 and PERK homodimerize upon release of Bip and undergo autophosphorylation. ATF6 is transported to the Golgi, where it is cleaved proteolytically. Activation of each sensor produces an active transcription factor, which in turn activates downstream target genes to restore ER homeostasis. Recently, *Brucella* infection was suggested to induce the UPR [13–15]. Qin et al. [13] demonstrated that *Brucella* replication is suppressed following the knockdown of IRE1 in insect cells and murine embryonic fibroblasts. De Jong et al. [14] suggested that *B*. *abortus* infection activated the IRE1 pathway, whereas Smith et al. [15] showed that all three UPR pathways were induced in infection of murine macrophages with *B*. *melitensis*. However, the precise role of the UPR in the intracellular life of *Brucella* spp., the host factors involved in replication processes, and the mechanism by which *Brucella* modulates the UPR remain unknown.

To gain a more comprehensive understanding of *Brucella*-host interaction, it is necessary to identify and characterize the host molecules involved in the biogenesis of replicative BCVs. Intracellular *Brucella* spp. secretes effector molecules into the host cytoplasm or onto the BCV membrane through a unique secretion system, and modulates intracellular trafficking to establish a safe replication niche. To date, several *Brucella* effectors have been reported. VceA and VceC are translocated into the host cytoplasm [16], and VceC triggers a host inflammatory response by inducing UPR-dependent NF-κB signaling [14]. RicA (Rab2 interacting conserved protein A) interacts with host Rab2, and affects the trafficking of BCVs [17]. CstA (conserved Sec24A-targeted protein A) interacts with Sec24A [18], whereas BspA, BspB, and BspF are targeted to the compartments of the secretory pathway [19]. TcpB (TIR domain containing-protein B) induces the upregulation of UPR target genes and structural reorganization of the ER [15]. However, the precise molecular functions of these *Brucella* effectors in replication still remain poorly characterized. Host factors that are involved in the ER-Golgi vesicular transport pathways, such as Sar1 [3], Rab2, and glyceraldehyde-3-phosphate dehydrogenase (GAPDH) [20] have been shown to be required for intracellular replication of *B*. *abortus*. The bacteria exploit Sar1 at ERES for BCVs to fuse with the ER [3]. GAPDH and Rab2 are recruited onto BCV membranes, which indicates that BCVs intercept retrograde trafficking and interact with the ER-Golgi intermediate compartment (ERGIC) [20]. Despite these previous studies, the molecular mechanisms for the biogenesis of replicative BCVs are still obscure. The way in which host factors contribute to *Brucella* intracellular life remains speculative. Identification of the host factors that are essential for the formation of ER-derived replicative BCVs and the characterization of their functions would help in elucidating these mechanisms. In the present study, we investigated a potential role of the UPR in *Brucella* intracellular life, and found that Yip1A, which regulates the activation of IRE1, is a pivotal host factor for *B*. *abortus* to establish its ER-derived safe replication niche.

## Results

### Infection with *Brucella abortus* activates the IRE1 pathway of the UPR and leads to the upregulation of the COPII vesicle components Sar1, Sec23 and Sec24D

First we monitored the intracellular replication of *B*. *abortus* in HeLa cells. At 24 hr post infection (p.i.), a significant increase in the number of colony forming units (CFUs) was observed (S1A Fig), and extensive intracellular replication was identified by immunofluorescence microscopy (S1B Fig).

**Fig 1:**
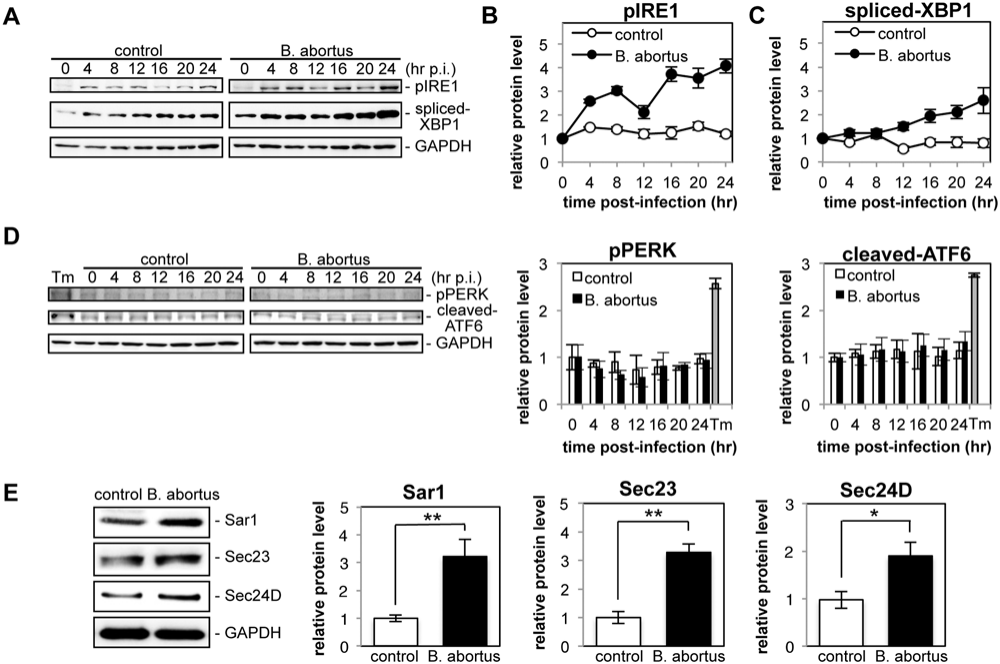
Infection with *Brucella abortus* activates the IRE1 pathway of the UPR and leads to the upregulation of the COPII vesicle components Sar1, Sec23 and Sec24D. HeLa cells were uninfected (‘control’) or infected with *B*. *abortus* (‘B. abortus’). Cell lysates were collected at the indicated time points and analyzed by Western blotting. (A) Representative immunoblots for pIRE1, spliced-XBP1, and GAPDH. GAPDH was used for normalization. The intensity of the bands was quantified using the MultiGauge software. (B, C) Relative protein levels of pIRE1 (B) and spliced-XBP1 (C) in uninfected control (open circles) and *Brucella*-infected (solid circles) cells. The protein levels at time 0 hr were assigned the value 1. Data are means ± SD from three independent experiments. (D) Representative immunoblots for pPERK, cleaved-ATF6, and GAPDH, and relative protein levels of pPERK and cleaved-ATF6 in control (open bars) and *Brucella*-infected (solid bars) cells. GAPDH was used for normalization. The intensity of the bands was quantified using the MultiGauge software, and the results are shown in the bar graphs. As a positive control for activation of PERK or ATF6, HeLa cells were treated with 5 μg/ml of tunicamycin for 8hr (‘Tm’). The protein levels at time 0 hr were assigned the value 1. Data are means ± SD from three independent experiments. (E) Representative immunoblots for Sar1, Sec23, Sec24D, and GAPDH, and relative protein levels of Sar1, Sec23, and Sec24D in control (open bars) and *Brucella*-infected (solid bars) cells. GAPDH was used for normalization. The intensity of the bands was quantified using the MultiGauge software, and the results are shown in the bar graphs. The protein levels in control cells were assigned the value 1. Data are means ± SD from three independent experiments. *: p<0.05; **: p<0.01.

To investigate the induction of the UPR during *Brucella* infection, HeLa cells were infected or not with *B*. *abortus*, and the activation of three UPR sensors (IRE1, PERK, and ATF6) was analyzed by Western blotting (Fig. 1, A and D). As shown by the increase in phosphorylated IRE1 (pIRE1), *Brucella* infection triggered the activation of IRE1 (Fig. 1B). At early time points (4 hr and 8 hr p.i.), and then later (16 hr p.i. onwards), a drastic increase in pIRE1 was observed in *B*. *abortus*-infected cells. Phosphorylated IRE1 removes a short intron from XBP1 mRNA, which results in the production of spliced-XBP1 protein [21]. As shown in Fig. 1C, spliced-XBP1 increased over time during *Brucella* infection. In contrast, the amount of phosphorylated PERK (pPERK) and cleaved-ATF6 was fairly constant over time both in control cells and in infected cells (Fig. 1D), which indicated that the PERK and ATF6 pathways were not activated by *Brucella* infection.

Activation of the IRE1 pathway upregulates an array of downstream target genes that are involved in the early secretory pathway [22]. Of those examined, we found that the expression of Sar1, Sec23, and Sec24D was enhanced significantly at 24 hr p.i. in *Brucella*-infected cells (Fig. 1E). These molecules are all involved in the formation of COPII vesicles at ERES [23].

These results demonstrate that infection with *B*. *abortus* preferentially activates the IRE1 pathway of the UPR, but not the PERK and ATF6 pathways, in HeLa cells, which leads to the upregulation of the COPII vesicle components Sar1, Sec23 and Sec24D.

### Yip1A interacts with pIRE1 at ERES

**Fig 2:**
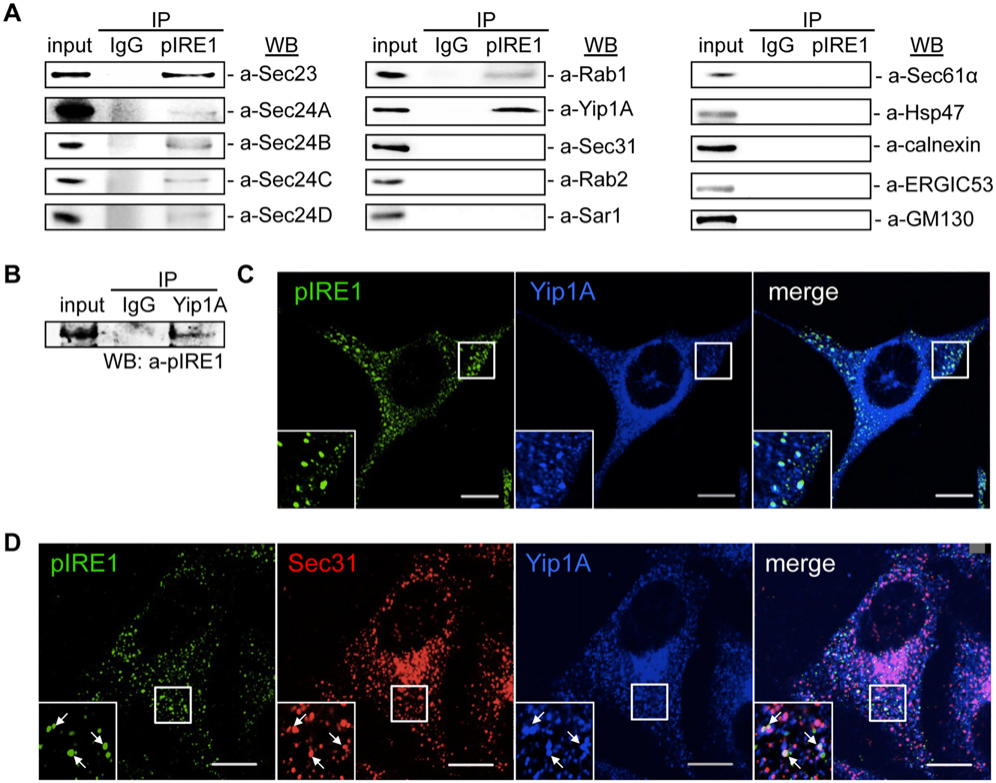
Yip1A interacts with pIRE1 at ERES. (A) Representative immunoblots showing co-immunoprecipitation with pIRE1. After 5 hr of Tm treatment, immunoprecipitation was performed on HeLa cell lysates with an anti-pIRE1 antibody (lanes labeled ‘pIRE1’) or control anti-rabbit IgG (lanes labeled ‘IgG’), and the immunoprecipitates were analyzed by Western blotting with a panel of antibodies against molecules involved in the ER-Golgi vesicular transport pathways or against ER- (Sec61α, HSP47, and calnexin), ERGIC- (ERGIC53) and *cis*-Golgi- (GM130) resident proteins. (B) Representative immunoblot showing the co-immunoprecipitation of pIRE1 with Yip1A. Following 5 hr of Tm treatment, immunoprecipitation was performed on HeLa cell lysates with an anti-Yip1A antibody (lane labeled ‘Yip1A’) or control anti-rabbit IgG (lane labeled ‘IgG’), and the immunoprecipitates were analyzed by Western blotting with an anti-pIRE1 antibody. (C) Representative confocal micrographs of HeLa cells double-stained for pIRE1 (green) and Yip1A (blue) after 5 hr of Tm treatment. Insets are magnifications of the boxed areas on the main image. Co-localized proteins were detected as large foci. Scale bars are 10 μm. (D) Representative confocal micrographs of HeLa cells triple-stained for pIRE1 (green), Sec31 (red), and Yip1A (blue) after 5 hr of Tm treatment. Insets are magnifications of the boxed areas on the main image. Co-localized proteins were identified as large, bright foci (arrows). Scale bars are 10 μm.

Functional ERES and COPII vesicles have been implicated in the intracellular replication of *B*. *abortus* [3]. The upregulation of the COPII vesicle components Sar1, Sec23 and Sec24D that follows the activation of the IRE1 pathway of the UPR (Fig. 1E) suggested that a host factor that links the UPR and COPII vesicle biogenesis may play an important role in the intracellular replication of *B*. *abortus*. To search for such a host factor, we performed an immunoprecipitation (IP) assay using an anti-pIRE1 antibody against HeLa cells that were treated with tunicamycin (Tm) to induce the UPR. The immunoprecipitates were analyzed by Western blotting for a panel of molecules known to be involved in the ER-Golgi vesicular transport pathways (Fig. 2A). Intriguingly, inner components of the COPII coat (Sec23, Sec24A, Sec24B, Sec24C and Sec24D), Rab1, and Yip1A (YPT-interacting protein 1A, also known as YIPF5) were found to interact with pIRE1. In contrast, a component of the outer coat (Sec31A), Sar1, Rab2, as well as some ER- (Sec61α, HSP47 and calnexin), ERGIC- (ERGIC53) and *cis*-Golgi- (GM130) resident proteins showed no specific interaction with pIRE1 (Fig. 2A).

Among the test panel, Yip1A, which is a 257 amino acid multi-pass membrane protein, was included as a candidate interacting partner for pIRE1, because it localizes to ERES, binds to Sec23 and Sec24, and is involved in COPII vesicle budding [24]. To the best of our knowledge, Yip1A had not previously been implicated in the intracellular replication of *B*. *abortus* or in the UPR, which prompted us to focus on this protein. To further confirm the specificity of the interaction between Yip1A and pIRE1, the IP assay was repeated but with an anti-Yip1A antibody, and pIRE1 was identified to bind to Yip1A (Fig. 2B). The interaction of Yip1A with pIRE1 was enhanced upon Tm treatment (S2 Fig), and thus dependent on the induction of the UPR.

Under the UPR condition, IRE1 molecules cluster into oligomers, and undergo trans-autophosphorylation [25, 26]. Accordingly, pIRE1 can be detected as large foci with an anti-pIRE1 antibody by immunofluorescence microscopy. After Tm treatment, these large pIRE1 foci indeed co-localized with Yip1A (Fig. 2C). Given that the foci were co-stained with Sec31, a marker for ERES (Fig. 2D), Yip1A and pIRE1 were located at ERES upon the induction of the UPR.

### Yip1A is responsible for the phosphorylation of IRE1 via the high-order assembly of IRE1 molecules

**Fig 3:**
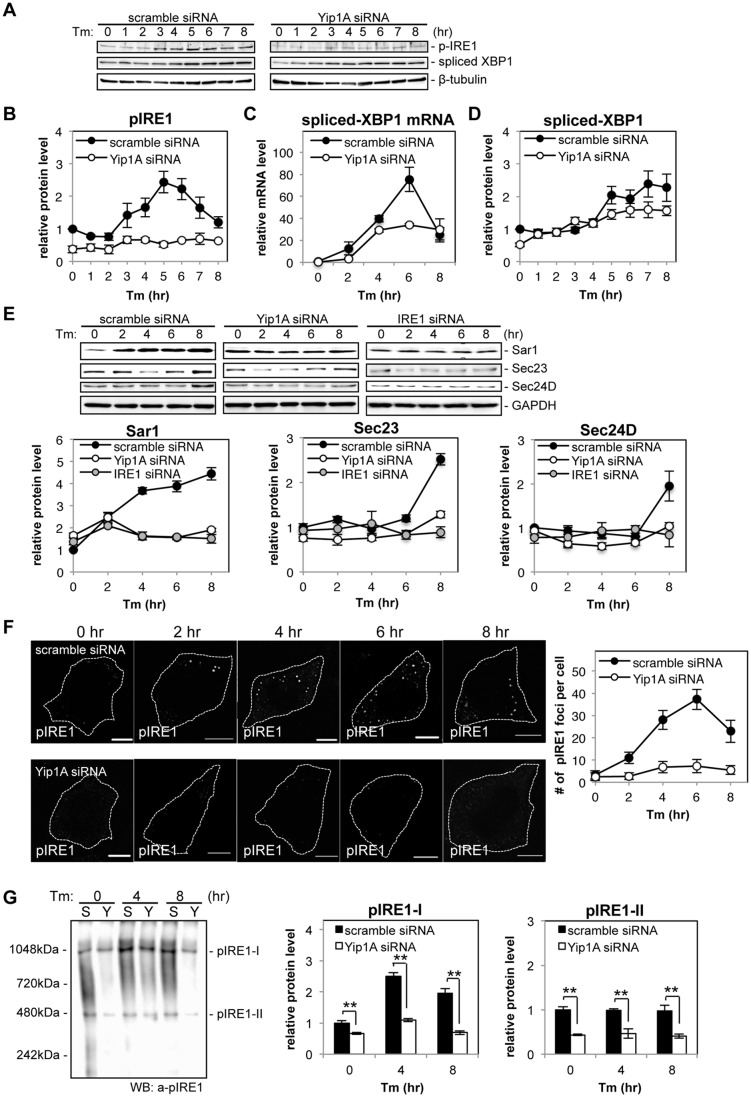
Yip1A is responsible for the phosphorylation of IRE1 via high-order assembly of Ire1 molecules. HeLa cells were transfected with each siRNA for 24 hr, and then treated with Tm to induce the UPR. Cell lysates were prepared at the indicated time points and analyzed by Western blotting. (A) Representative immunoblots for pIRE1, spliced-XBP1, and β-tubulin. β-tubulin was used for normalization. The intensity of the bands was quantified using the MultiGauge software. (B-D) Relative protein levels of pIRE1 (B), relative mRNA levels of spliced-XBP1 (C), and relative protein levels of spliced-XBP1 (C) in control (solid circles) and Yip1A-knockdown (open circles) cells. The protein or mRNA levels in control cells at the beginning of the Tm treatment were assigned the value 1. Data are means ± SD from three independent experiments. (E) Representative immunoblots for Sar1, Sec23, Sec24D, and GAPDH, and relative protein levels of Sar1, Sec23, and Sec24D in control (solid circles), Yip1A-knockdown (open circles), and IRE1-knockdown (solid gray circles) cells. GAPDH was used for normalization. The intensity of the bands was quantified using the MultiGauge software, and the results are shown in the line graphs. The protein levels in control cells at the beginning of the Tm treatment were assigned the value 1. Data are means ± SD from three independent experiments. (F) Representative confocal micrographs of control (upper panels) or Yip1A-knockdown (lower panels) cells during the Tm treatment. Fixed cells at the indicated time points were stained for pIRE1. Cells are outlined with white dashed lines. Scale bars are 10 μm. The numbers of pIRE1 foci per cell were counted, and shown in the line graph. Data are means ± SD (N = 30). (G) Representative immunoblot for pIRE1 after native PAGE, showing two high-order complexes of pIRE1 (pIRE1-I and pIRE1-II). Lane ‘S’ represents lysate from HeLa cells transfected with control scramble siRNA, and lane ‘Y’ represents lysate from HeLa cells transfected with Yip1A siRNA. Numbers on the left-hand side correspond to the standard molecular weight. The intensity of the bands was quantified by using the MultiGauge software, and the results are shown in the bar graphs. Protein levels in control cells at the beginning of the Tm treatment were assigned the value 1. Data are means ± SD from three independent experiments. **: p<0.01.

Next, we knocked down the expression of Yip1A in HeLa cells by using small interfering RNA (siRNA) to determine whether Yip1A localized at ERES is involved in the activation of IRE1. The expression of Yip1A was reduced by 72.5% (S3A Fig). The knockdown of Yip1A was further confirmed by immunofluorescence microscopy (S3B Fig). The cells transfected with siRNA were then treated with Tm to induce the UPR, and activation of the IRE1 pathway was analyzed by Western blotting (Fig. 3A). There was no difference in the total levels of IRE1 between control and Yip1A-knockdown cells throughout the experiment (S3C Fig). In control cells, the phosphorylation of IRE1 peaked at 5 hr after the addition of Tm, and then began to decrease (Fig. 3B). The splicing of XBP1 mRNA correlated with the activation of IRE1 (Fig. 3C), which resulted in an increase in spliced-XfBP1 protein from 5 hr onwards (Fig. 3D). Strikingly, knockdown of Yip1A diminished the increase in pIRE1 throughout the course of Tm treatment (Fig. 3B). Consistent with this result, the splicing of XBP1 mRNA (Fig. 3C) and the amount of spliced-XBP1 protein (Fig. 3D) were reduced by the depletion of Yip1A. In contrast, Yip1A knockdown had no effect on the activation of PERK or ATF6 during Tm treatment (S3D Fig). During infection with *B*. *abortus*, the downstream targets of the IRE1 pathway Sar1, Sec23, and Sec24D, were upregulated significantly (Fig. 1E). In control cells, Tm treatment also increased the amounts of these molecules, whereas Yip1A-knockdown cells showed little upregulation of them (Fig. 3E). In addition, knockdown of IRE1 caused similar results to those of Yip1A-knockdown (Fig. 3E). The expression of IRE1 was reduced by 84.3% (S3E Fig).

To examine the effect of Yip1A-knockdown on the localization of total IRE1, immunofluorescence microscopy was performed after 5 hr of Tm treatment (S3F Fig). Several large vacuoles were observed in control cells (S3F Fig, left-hand panels, arrows), but not in Yip1A-knockdown cells (S3F Fig, right-hand panel); otherwise, IRE1 was stained throughout the cytoplasm in a reticular pattern both in control and in Yip1A-knockdown cells, indicating its intrinsic localization in the ER. We therefore concluded that the localization of IRE1 was not affected by Yip1A-knockdown. Then, we hypothesized that the deficiency in IRE1 phosphorylation caused by Yip1A-knockdown may be attributed to the inability of IRE1 to form oligomers. To examine this possibility, the formation of large pIRE1 foci was assessed by immunofluorescence microscopy under Tm treatment (Fig. 3F). HeLa cells were transfected with scramble siRNA or Yip1A siRNA for 24 hr, and then treated with Tm to induce the UPR. The number of pIRE1 foci per cell was counted in these cells. In control cells, time-dependent appearance of pIRE1 foci was observed: the number of foci increased during the first 6 hr of Tm treatment, and then started to decrease (Fig. 3F, upper panels), consistent with the result obtained in the Western blot analysis of pIRE1 (Fig. 3B). By contrast, in Yip1A knockdown cells, pIRE1 foci were hardly observed throughout the Tm treatment (Fig. 3F, lower panels). These results support the idea that IRE1 molecules fail to assemble into cluster under the UPR condition in the absence of Yip1A.

The above effect of Yip1A-knockdown on the oligomeric state of IRE1 was further demonstrated by native polyacrylamide gel electrophoresis (PAGE), which resolved pIRE1 molecules as two high-order complexes with apparent molecular weights of approximately 500kDa and 1000kDa (pIRE1-I and pIRE1-II, respectively) (Fig. 3G). In control cells, the amount of pIRE1-I was increased after 4 hr of Tm treatment and then decreased (Fig. 3G, lanes labeled ‘S’), coincide with the results of the Western blot analysis of pIRE1 (Fig. 3B) or the formation of pIRE1 foci (Fig. 3F). The amount of pIRE1-II remained constant throughout the Tm treatment. In Yip1A knockdown cells, the amount of both high-order complexes was reduced significantly (Fig. 3G, lanes labeled ‘Y’). Collectively, these data support the idea that Yip1A is responsible for the phosphorylation of IRE1 via the high-order assembly of IRE1 molecules under the UPR condition.

### Yip1A is involved in the formation of large vacuoles through the activation of IRE1, and Atg9 and WIPI1, but not DFCP1, are required for this process

**Fig 4:**
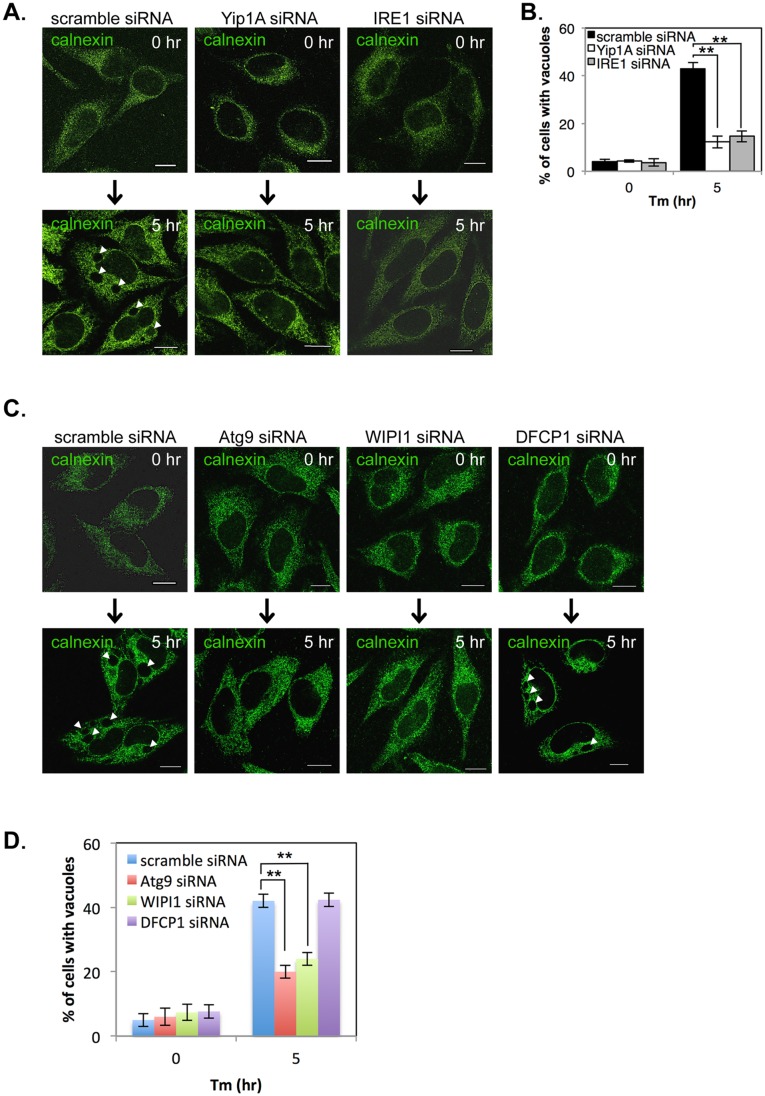
Yip1A is involved in the formation of large vacuoles through the activation of IRE1, and Atg9 and WIPI1, but not DFCP1, are required for this process. HeLa cells were transfected with each siRNA for 24 hr, and then treated with Tm for 5 hr to induce the UPR. (A, B) Representative confocal micrographs of control (left-hand panels), Yip1A-knockdown (middle panels), and IRE1-knockdown (right-had panels) cells after 0 hr or 5 hr of Tm treatment (A). The ER structure was visualized with an anti-calnexin antibody. Large vacuoles were observed in control cells (arrowheads), but not in Yip1A- or IRE1-knockdown cells. Scale bars are 10 μm. The percentage of cells with vacuoles was counted, and is shown in the bar graph (B). Data are means ± SD from three independent experiments (N = 100). **: p<0.01. (C, D) Representative confocal micrographs of control, Atg9-knockdown, WIPI1-knockdown, and DFCP1-knockdown cells after 0 hr or 5 hr of Tm treatment (C). The ER structure was visualized with an anti-calnexin antibody. Large vacuoles were observed in control and DFCP1-knockdown cells (arrowheads), but rarely seen in Atg9- or WIPI1-knockdown cells. Scale bars are 10 μm. The percentage of cells with vacuoles was counted, and is shown in the bar graph (D). Data are means ± SD from three independent experiments (N = 100). **: p<0.01.

During Tm treatment, large vacuoles were observed in control cells but not in Yip1A-knockdown cells (S3F Fig). We wondered whether vacuolization induced by Tm treatment is also dependent on the activation of IRE1 through Yip1A, and investigated the effect of Yip1A- or IRE1-knockdown on vacuolization under the UPR condition. HeLa cells were transfected with each siRNA and then treated with Tm to induce the UPR. The ER structure was visualized by immunofluorescence microscopy with an anti-calnexin antibody (Fig. 4A). Whereas large vacuoles were formed after Tm treatment in control cell (Fig. 4A, left-hand panels, arrows), such vacuolization was not seen in Yip1A-knockdown (Fig. 4A, middle panels) or IRE1-knockdown (Fig. 4A, right-hand panels) cells. The percentage of cells with vacuoles was significantly lower in Yip1A- or IRE1-knockdown cells than in control cells after Tm treatment (Fig. 4B). Thus, there is likely to be a link between Yip1A-mediated activation of IRE1 and the formation of large vacuoles under the UPR condition.

The activation of the UPR has been implicated in the induction of ER-derived autophagy [27, 28], and ERES plays a key role during formation of the isolation membrane (IM), the very early structure in autophagosome biogenesis [29–31]. In addition, the COPII vesicles budding from ERES have been recently shown to supply membrane for autophagosome formation [29, 32, 33]. On the basis of these findings, we assumed that the formation of large vacuoles might be related to an ER-derived autophagic event that is triggered by the UPR. To verify this, we examined the formation of large vacuoles using siRNA against the autophagy-related proteins Atg9, WIPI1 (WD repeat domain phosphoinositide-interacting protein 1; one of mammalian homologues of yeast Atg18), and DFCP1 (Double FYVE-containing protein 1, also known as ZFYVE 1 (zinc finger FYVE domain-containing protein 1)), all of which are known to translocate to the IM at an earlier stage of autophagosome biogenesis [34–36]. The expression of Atg9, WIPI1, and DFCP1 was reduced by about 80% at 24 hr after siRNA transfection (S4 Fig). HeLa cells were transfected with each siRNA, and then treated with Tm to induce the UPR. The ER structure was visualized by immunofluorescence microscopy with an anti-calnexin antibody (Fig. 4C). Notably, Atg9- or WIPI1-knockdown prevented the formation of large vacuoles, but knockdown of DFCP1 did not. The percentage of cells with vacuoles was significantly reduced in Atg9- or WIPI1-knockdown cells, as compared with control and DFCP1-knockdown cells after Tm treatment (Fig. 4D). These results indicate that the earlier autophagic process, which involves Atg9 and WIPI1, but not DFCP1, is likely to be required for formation of the large vacuoles during the UPR condition.

### Yip1A is required for the activation of the IRE1 pathway and the upregulation of Sar1, Sec23 and Sec24D during infection with *B*. *abortus*

**Fig 5:**
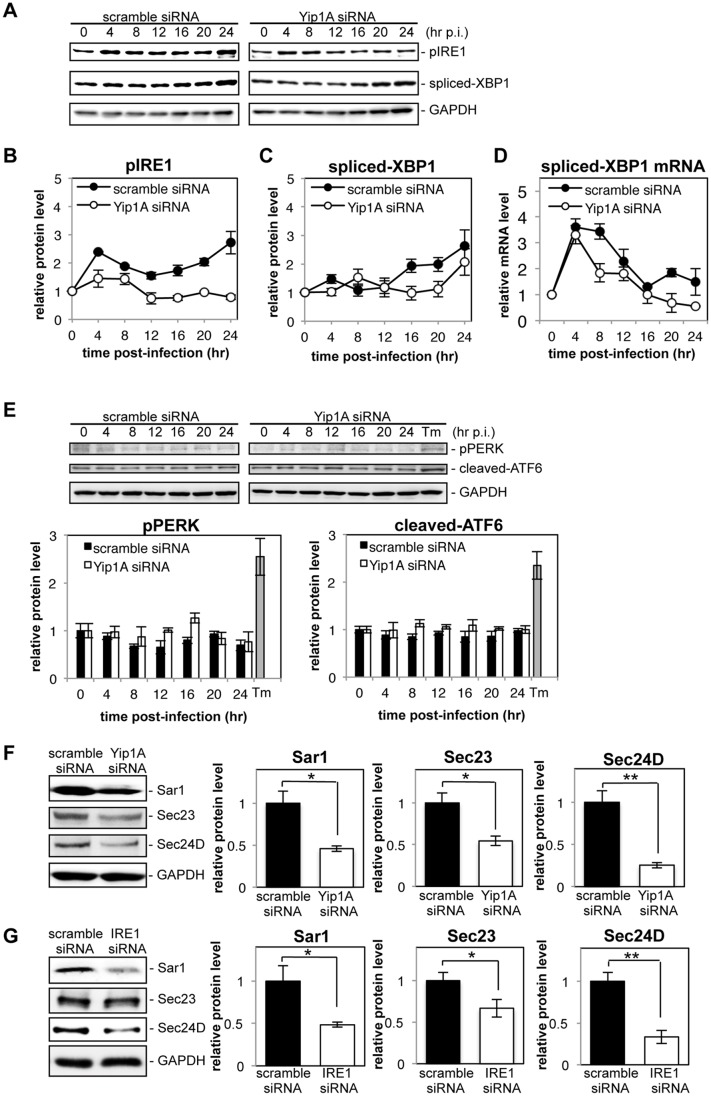
Yip1A is required for the activation of the IRE1 pathway and the upregulation of Sar1, Sec23 and Sec24D during infection with *B*. *abortus*. HeLa cells were infected with *B*. *abortus*, and then transfected with each siRNA at 1 hr p.i. Cell lysates were collected at the indicated time points, and analyzed by Western blotting. (A) Representative immunoblots for pIRE1, spliced-XBP1, and GAPDH. GAPDH was used for normalization. The intensity of the bands was quantified using the MultiGauge software. (B-D) Relative protein levels of pIRE1 (B) and spliced-XBP1 (C), and relative mRNA levels of spliced-XBP1 (D) in control (solid circles) and Yip1A-knockdown (open circles) cells. The protein levels at time 0 hr were assigned the value 1. Data are means ± SD from three independent experiments. (E) Representative immunoblots for pPERK, cleaved-ATF6, and GAPDH, and relative protein levels of pPERK and cleaved-ATF6 in control (solid bars) and Yip1A-knockdown (open bars) cells. GAPDH was used for normalization. The intensity of the bands was quantified using the MultiGauge software, and the results are shown in the bar graphs. As a positive control for activation of PERK or ATF6, HeLa cells were treated with 5 μg/ml tunicamycin for 8 hr (‘Tm’). The protein levels at time 0 hr were assigned the value 1. Data are means ± SD from three independent experiments. (F) Representative immunoblots for Sar1, Sec23, Sec24D, and GAPDH, and relative protein levels of Sar1, Sec23, and Sec24D in control (solid bars) and Yip1A-knockdown (open bars) cells at 24 hr p.i. GAPDH was used for normalization. The intensity of the bands was quantified using the MultiGauge software, and the results are shown in the bar graphs. The protein levels in control cells were assigned the value 1. Data are means ± SD from three independent experiments. *: p<0.05; **: p<0.01. (G) Representative immunoblots for Sar1, Sec23, Sec24D, and GAPDH, and relative protein levels of Sar1, Sec23, and Sec24D in control (solid bars) and IRE1-knockdown (open bars) cells at 24 hr p.i. GAPDH was used for normalization. The intensity of the bands was quantified using the MultiGauge software, and the results are shown in the bar graphs. The protein levels in control cells were assigned the value 1. Data are means ± SD from three independent experiments. *: p<0.05; **: p<0.01.

To determine whether Yip1A-knockdown has the same effects during infection with *B*. *abortus*, HeLa cells were transfected with scramble siRNA or Yip1A siRNA 1 hr after infection. Infection with *B*. *abortus* preceded the siRNA transfection to eliminate any effects of Yip1A knockdown on the internalization of *B*. *abortus*. Using RT-PCR, we confirmed that Yip1A mRNA was reduced by approximately 80% from 12 hr p.i. onwards (S5A Fig). A similar knockdown efficiency of Yip1A protein was achieved at 12 hr p.i. onwards (S5B Fig), and this knockdown of Yip1A protein was considered to be sufficient to demonstrate the role of Yip1A on the intracellular replication of *B*. *abortus* at these later time points. At 4 hr or 8 hr p.i., the knockdown of Yip1A protein was 17±11% or 46±8%, and thus the effects of Yip1A knockdown at these time points were likely to be limited. There was no difference in the total levels of IRE1 between control and Yip1A-knockdown cells throughout the experiment (S5C Fig). The activation of the UPR sensors IRE1, PERK, and ATF6 was analyzed by Western blotting (Fig. 5, A and E). Control cells showed activation kinetics for these molecules similar to those obtained in uninfected cells (Fig. 5, B and E). In Yip1A-knockdown cells, the increase in pIRE1 was partially suppressed at early time points (4 hr and 8 hr p.i.), which may reflect insufficient knockdown of Yip1A (S5B Fig), but was abolished completely at 12 hr p.i. onwards (Fig. 5B). The splicing of XBP1 appeared to be delayed in these cells (Fig. 5C). RT-PCR for spliced-XBP1 mRNA revealed the distinct splicing kinetics between control and Yip1A-knockdown cells more clearly (Fig. 5D). In control cells, the levels of spliced-XBP1 mRNA increased along with the increase in pIRE1: first at 4–8 hr p.i., and then at 20 hr p.i (Fig. 5D). In Yip1A-knockdown cells, the lack of IRE1 activation at later time points led to complete loss of spliced XBP1 mRNA. The levels of pPERK and cleaved ATF6 remained almost the same between control and Yip1A-knockdown cells during infection (Fig. 5E). These results support the significance of Yip1A in the activation of the IRE1 pathway during infection with *B*. *abortus*.

Next, we investigated the effects of Yip1A-knockdown on the upregulation of the COPII components Sar1, Sec23, and Sec24D. At 24 hr p.i., the levels of Sar1, Sec23, and Sec24D were significantly lower in Yip1A-knockdown cells than in control cells (Fig. 5F). The levels of these proteins were reduced to approximately the same levels observed in uninfected cells, except for Sec24D, which was reduced to 75% of the levels in uninfected cells (S5D Fig). To demonstrate the functional connection between Yip1A and IRE1 in terms of these results, expression of IRE1 was knocked down by using siRNA. Marked depletion of IRE1 was observed at 12 hr p.i. onwards (S5E Fig). The levels of Sar1, Sec23 and Sec24D were similarly diminished in IRE1-knockdown cells (Fig. 5G).

### Yip1A-knockdown results in deficient *B*. *abortus* replication within HeLa cells

**Fig 6:**
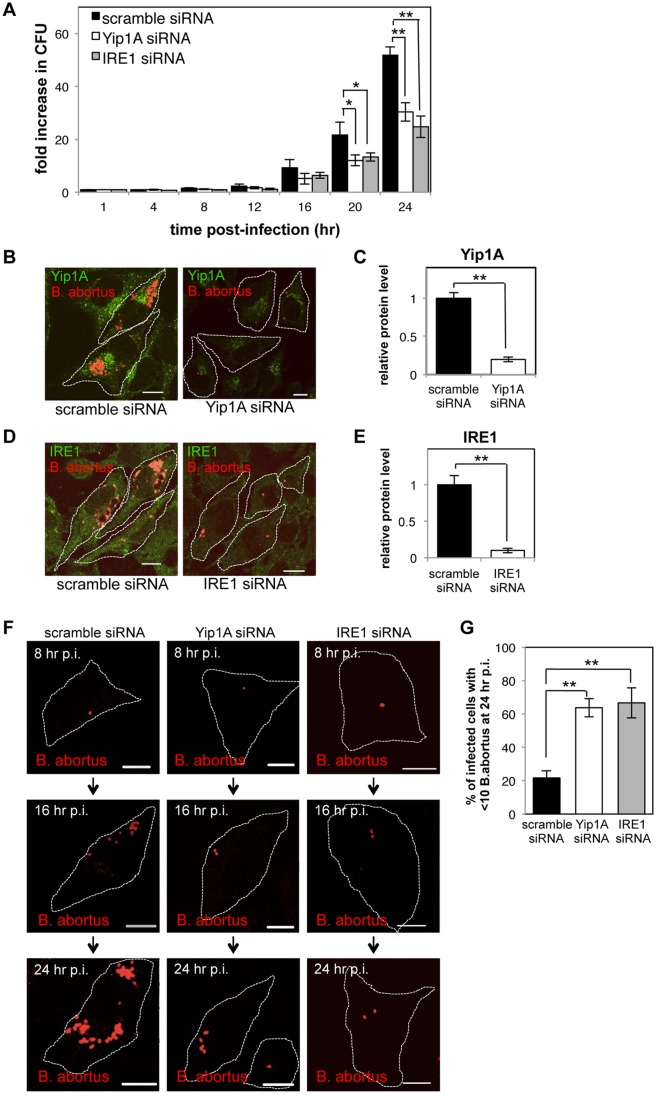
Yip1A-knockdown results in deficient replication of *B*. *abortus* within HeLa cells. HeLa cells were infected with *B*. *abortus*, and then transfected with each siRNA at 1 hr p.i. (A) Intracellular growth of *B*. *abortus* within control (solid bars), Yip1A-knockdown (open bars), and IRE1-knockdown (solid gray bars) cells. CFUs were enumerated at the indicated time points. Data are means ± SD from three independent experiments. *: p<0.05; **: p<0.01. (B, C) Representative confocal micrographs of control (left-hand panel) and Yip1A-knockdown (right-hand panel) cells at 24 hr p.i. (B). Fixed cells were double-stained for Yip1A (green) and *B*. *abortus* (red). The infected cells are outlined with white dashed lines. Scale bars are 10 μm. The knockdown efficiency of Yip1A in infected cells was evaluated by quantifying the intensity of immunofluorescence staining for Yip1A, and the result is shown in the bar graph (C). (D, E) Representative confocal micrographs of control (left-hand panel) and IRE1-knockdown (right-hand panel) cells at 24 hr p.i. (D). Fixed cells were double-stained for IRE1 (green) and *B*. *abortus* (red). The infected cells are outlined with white dashed lines. Scale bars are 10 μm. The knockdown efficiency of IRE1 in infected cells was evaluated by quantifying the intensity of immunofluorescence staining for IRE1, and the result is shown in the bar graph (E). (F, G) Representative confocal micrographs of control (left-hand panels), Yip1A-knockdown (middle panels), and IRE1-knockdown (right-hand panels) cells at 8 hr, 16 hr, and 24 hr p.i. (F). Fixed cells were stained for *B*. *abortus*. The infected cells are outlined with white dashed lines. Scale bars are 10 μm. To assess the replication efficiency, the percentage of infected cells with fewer than ten *B*. *abortus* was determined at 24hr p.i., and the result is shown in the bar graph (G).

We investigated the effects of Yip1A knockdown on the intracellular replication of *B*. *abortus*. First, intracellular bacterial growth was evaluated by counting CFUs over 24 hr. The kinetics of *Brucella* replication in control cells agreed with those obtained in previous studies [4, 6] (Fig. 6A, black bars). Intriguingly, Yip1A knockdown inhibited bacterial growth, which resulted in about a 40% reduction in CFUs at 24 hr p.i. (Fig. 6A, white bars). Here again, IRE1-knockdown suppressed the increase in CFU similar to Yip1A-knockdown, and caused about a 50% reduction in CFU at 24 hr p.i. (Fig. 6A, gray bars).

To confirm further the effect on intracellular replication, we examined the number of *B*. *abortus* within infected cells by using immunofluorescence microscopy. The knockdown efficiency of Yip1A or IRE1 in infected cells was evaluated by quantifying the intensity of immunofluorescence staining for endogenous Yip1A (Fig. 6B) or IRE1 (Fig. 6D) at 24 hr p.i., which showed approximately 80% and 90% of depletion had been achieved for Yip1A (Fig. 6C) and IRE1 (Fig. 6E), respectively. In control cells, the onset of bacterial replication could be seen at 16 hr p.i., and the cytoplasm of an infected cell was filled with robustly replicating bacteria at 24 hr p.i. (Fig. 6, B and D, left-hand panels; and Fig. 6F, left-hand panels). In contrast, Yip1A-knockdown cells (Fig. 6B, right-hand panel; and Fig. 6F, middle panels) or IRE1-knockdown cells (Fig. 6D, right-hand panel; and Fig. 6F, right-hand panels) contained a considerably small number of *B*. *abortus* at 24 hr p.i. To assess the replication efficiency, the percentage of infected cells with fewer than ten *B*. *abortus* was determined at 24 hr p.i. As can be seen in Fig. 6G, Yip1A- or IRE1-knockdown significantly suppressed the intracellular replication of *B*. *abortus*. Altogether, these results indicate that the activation of the IRE1 pathway is critical to establish a safe replication niche, and that Yip1A is indispensable for this process during infection with *B*. *abortus*.

### Yip1A-knockdown prevents maturation of *B*. *abortus* into ER-derived replicative BCVs, and confines BCVs within Lamp2-positive compartments

**Fig 7:**
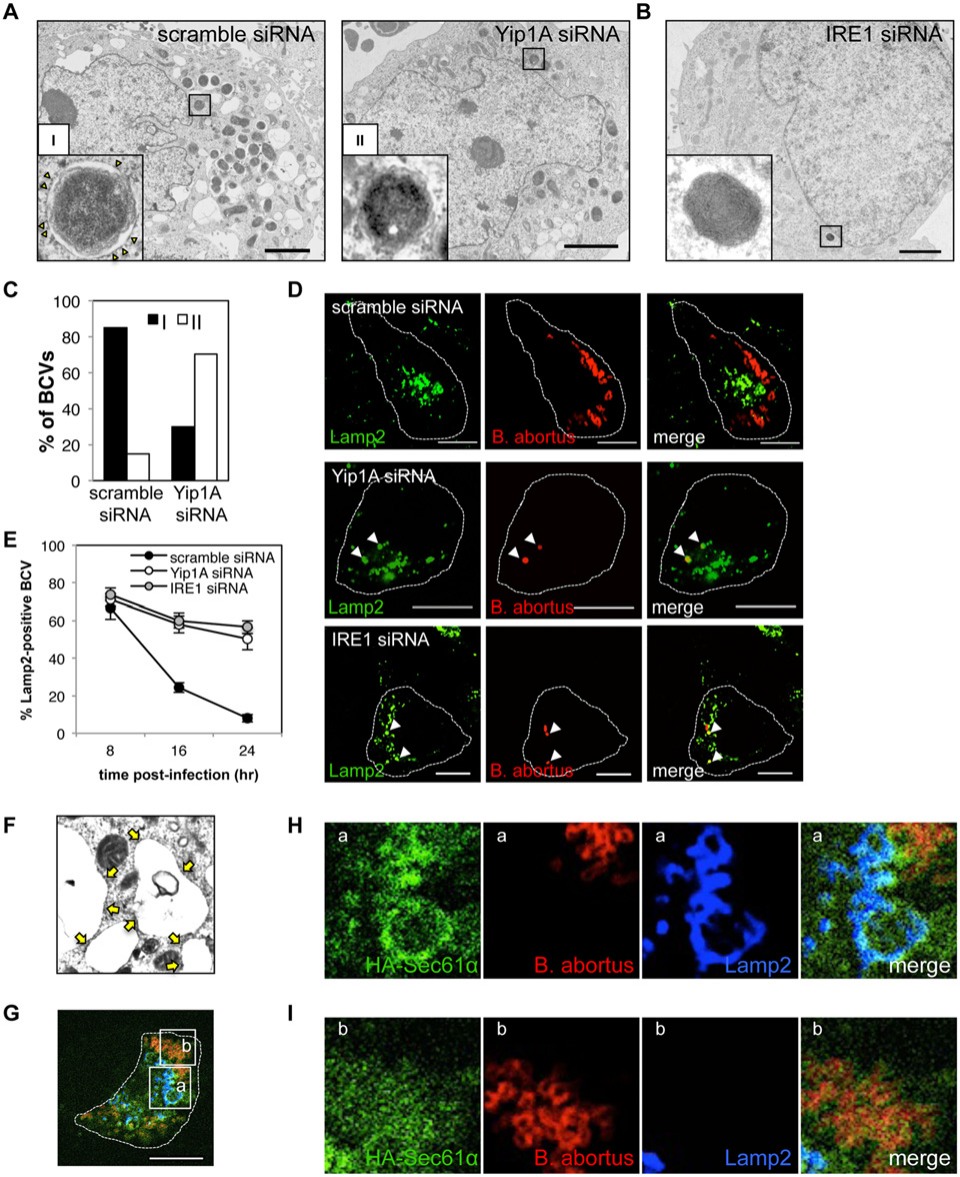
Yip1A-knockdown prevents maturation of *B*. *abortus* into ER-derived replicative BCVs, and confines BCVs within Lamp2-positive compartments. (A) Representative electron micrographs of *Brucella*-infected control (left-hand panel) and Yip1A-knockdown (right-hand panel) cells at 24 hr p.i. Insets are magnifications of the boxed areas on the main image, showing the typical forms of BCVs. In control cells, BCVs can be seen in the form of ER-derived membrane-bound compartments (inset in left-hand panel, defined as ‘I’). Note the presence of ribosomes on the membrane (arrowheads). In Yip1A-knockdown cells, the bacteria were not sequestered into such compartments (inset in right-hand panel, defined as ‘II’). Scale bars are 2 μm. (B) Representative electron micrograph of *Brucella*-infected IRE1-knockdown cells at 24 hr p.i. Inset is a magnification of the boxed area in the main image, showing that the bacteria were not sequestered into ER-derived membrane. Scale bar is 2 μm. (C) The percentages of the two forms of BCVs (I and II) present in control and Yip1A-knockdown cells at 24 hr p.i. The total numbers of BCVs analyzed were 67 for control cells and 37 for Yip1A-knockdown cells. (D, E) Representative confocal micrographs of control (upper panels), Yip1A-knockdown (middle panels), and IRE1-knockdown (lower panels) cells double-stained for Lamp2 (a marker for late endosomes/lysosomes; green) and *B*. *abortus* (red) at 24 hr p.i. BCVs co-localized with Lamp2 are indicated by arrowheads. The infected cells are outlined with white dashed lines. Scale bars are 10 μm. The percentage of Lamp2-positive BCVs was determined, and is shown in the line graph (E). (F) Magnification of the electron micrograph of the *Brucella*-infected control cell in (A), showing large vacuoles studded locally with ribosomes (arrows). (G-I) Representative confocal micrographs of *Brucella*-infected control cells at 24 hr p.i. triple-stained for HA-Sec61α (a marker for rough ER; green), *B*. *abortus* (red), and Lamp2 (a marker for endosomes/lysosomes; blue) (G). An expression construct for HA-Sec61α was co-transfected with scramble siRNA. The infected cell is outlined with white dashed lines. Scale bars are 10 μm. Magnifications of the boxed areas ‘a’ and ‘b’ are shown in (H) and (I), respectively. Large vacuoles adjacent to replicating bacteria were stained for both Sec61α and Lamp2 (H). *B*. *abortus* was co-stained with Sec61α but not with Lamp2 (I).

To characterize the deficiency in intracellular replication observed in Yip1A- or IRE1-knockdown cells, we performed an ultrastructural analysis of BCVs by electron microscopy (EM). Fig. 7A shows the electron micrographs obtained at 24 hr p.i. In control cells, infection with *B*. *abortus* generated a significant number of replicative BCVs with vacant vacuoles in their vicinity (Fig. 7A, left-hand panel). These membrane-bound compartments were derived from the ER, because ribosomes lined their surface (Fig. 7A, left-hand panel, inset). The lumens of the vacuoles were dilated, which resulted in massive ER expansion. As compared with control cells, Yip1A-knockdown cells displayed distinct morphological features (Fig. 7A, right-hand panel). Only a few bacteria were observed within the cells, and enlarged vacuoles were rarely seen. Notably, most BCVs were not enclosed in ER-derived membranes (Fig. 7A, right-hand panel, inset). Similar results were obtained in IRE1-knockdown cells (Fig. 7B). Thus, the EM analysis of infected cells revealed two forms of BCVs, one with an outermost ER-derived membrane (Fig. 7A, left-hand panel, inset; defined as I), and the other devoid of the ER-derived membrane (Fig. 7A, right-hand panel, inset; defined as II). At 24 hr p.i., 85% of BCVs in control cells had acquired the ER-derived membrane, whereas 70% of BCVs in Yip1A-knockdown cells were not sequestered into such a membrane (Fig. 7C).

To investigate the intracellular trafficking of BCVs in Yip1A knockdown cells, we performed immunofluorescence microscopy for Lamp2, a marker for late endosomes/lysosomes, and co-localization of BCVs with Lamp2-positive vacuoles was assessed over time (Fig. 7, D and E). In control cells, BCVs left Lamp2-positive compartments in a time-dependent manner, and 92% of BCVs were Lamp2-negative at 24 hr p.i. By contrast, about 50% of BCVs were co-localized with Lamp2 in Yip1A-knockdown cells (Fig. 7E), suggesting that these BCVs were confined within endosomal/lysosomal compartments. Interestingly, IRE1-knockdown cells showed kinetics similar to those of Yip1A-knockdown cells. Taken together, these results strongly suggest that *B*. *abortus* induces a marked accretion of ER-derived vacuoles around replicating bacteria to mature into ER-derived replicative BCVs, and that the activation of IRE1, which is mediated by Yip1A, is required for this process.

The vacant vacuoles adjacent to replicating bacteria appeared to be studded with ribosomes (Fig. 7, A and F). To further confirm the ER feature on these vacuoles, we performed immunofluorescence microscopy (Fig. 7, G-I). The vacant vacuoles were stained for both Lamp2 (a marker for endosomes/lysosomes) and Sec61α (a marker for rough ER), attesting to both the endosomal/lysosomal and ER-derived origin of these compartments (Fig. 7H). This indicates that the formation of ER-derived vacuoles and subsequent fusogenic events with Lamp2-positive compartments are likely to occur in *Brucella-*infected cells. The bacteria were co-stained with Sec61α but not with Lamp2 (Fig. 7I), consistent with the transition from endosomal/lysosomal to ER-derived BCVs.

### Atg9 and WIPI1, but not DFCP1, are required for the generation of replicative BCVs

**Fig 8:**
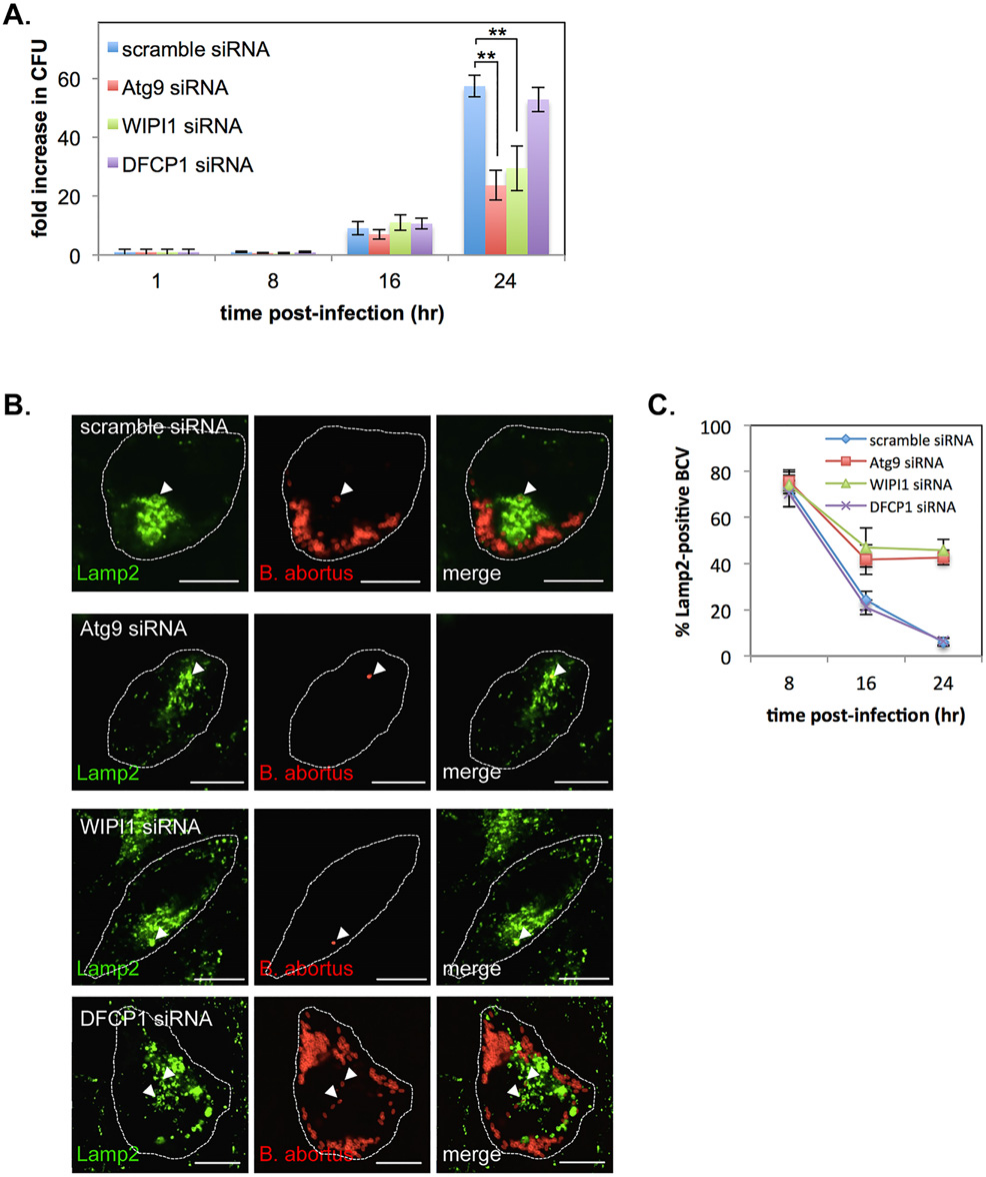
Atg9- and WIPI1, but not DFCP1, are required for the generation of replicative BCVs. HeLa cells were infected with *B*. *abortus*, and then transfected with each siRNA at 1 hr p.i. (A) Intracellular growth of *B*. *abortus* within control (blue bars), Atg9-knockdown (red bars), WIPI1-knockdown (green bars), and DFCP1-knockdown (purple bars) cells. CFUs were counted at the indicated time points after infection. Data are means ± SD from three independent experiments. **: p<0.01. (B, C) Representative confocal micrographs of control (top row), Atg9-knockdown (second row), WIPI1-knockdown (third row), and DFCP1-knockdown (bottom row) cells double-stained for Lamp2 (green) and *B*. *abortus* (red) at 24 hr p.i. BCVs co-localized with Lamp2 are indicated by arrowheads. The infected cells are outlined with white dashed lines. Scale bars are 10 μm. The percentage of Lamp2-positive BCVs was determined, and is shown in the line graph (C).

In the present study, we found that Yip1A is involved in the formation of large vacuoles via the activation of IRE1 under the UPR condition (Fig. 4, A and B), and that the formation of these vacuoles depends on Atg9 and WIPI1, but not DFCP1 (Fig. 4, C and D). We then tested whether these autophagy-related proteins were associated with the biogenesis of replicative BCVs by using siRNA. HeLa cells were infected with *B*. *abortus*, and transfected with each siRNA at 1hr p.i. Marked depletion of these proteins was achieved at 16 hr p.i. onwards (S6 Fig). First, we evaluated intracellular bacterial growth by counting CFUs over 24 hr. Intriguingly, Atg9- or WIPI1-knockdown significantly inhibited bacterial growth, resulting in about a 60% or 50% reduction in CFUs, respectively, at 24 hr p.i. (Fig. 8A). In contrast, little effect on bacterial growth was observed in DFCP1-knockdown cells. Then, we examined the trafficking of *B*. *abortus* in these knockdown cells using immunofluorescence microscopy for Lamp2 (Fig. 8, B and C). Co-localization of BCVs with Lamp2-positive vacuoles was monitored over time. In DFCP1-knockdown cells, BCVs followed kinetics similar to those of control cells, and about 94% of BCVs were Lamp2-negative at 24 hr p.i. In Atg9- or WIPI1-knockdown cells, by contrast, about 43% or 46% of BCVs were still within Lamp2-positive endosomal/lysosomal compartments, respectively (Fig. 8C). These results indicate that Atg9 and WIPI1, but not DFCP1, are required for the generation of replicative BCVs.

## Discussion

*Brucella* spp. replicates within an ER-derived membrane-bound compartment in host cells. However, the molecular mechanisms by which the pathogen establishes the replicative niche remain unclear. In the present study, we demonstrated several lines of evidence that clarify the mechanism by which *B*. *abortus* acquires the ER-derived membrane. First, during *Brucella* infection, the IRE1 pathway, but not the PERK and ATF6 pathways, of the UPR was activated, and the COPII vesicle components Sar1, Sec23, and Sec24D were upregulated. Second, biogenesis of ER-derived vacuoles was observed in the close proximity of replicating bacteria in *Brucella*-infected cells. Third, we identified Yip1A as a novel host factor that is required for the activation of IRE1 and the subsequent formation of ER-derived vacuoles. In Yip1A-knockdown cells, *B*. *abortus* failed to be sequestered within ER-derived membrane, and remained in an endosomal/lysosomal compartments. Furthermore, we found that the autophagy-related proteins Atg9- and WIPI1, but not DFCP1, are required for the biogenesis of replicative BCVs.

**Fig 9:**
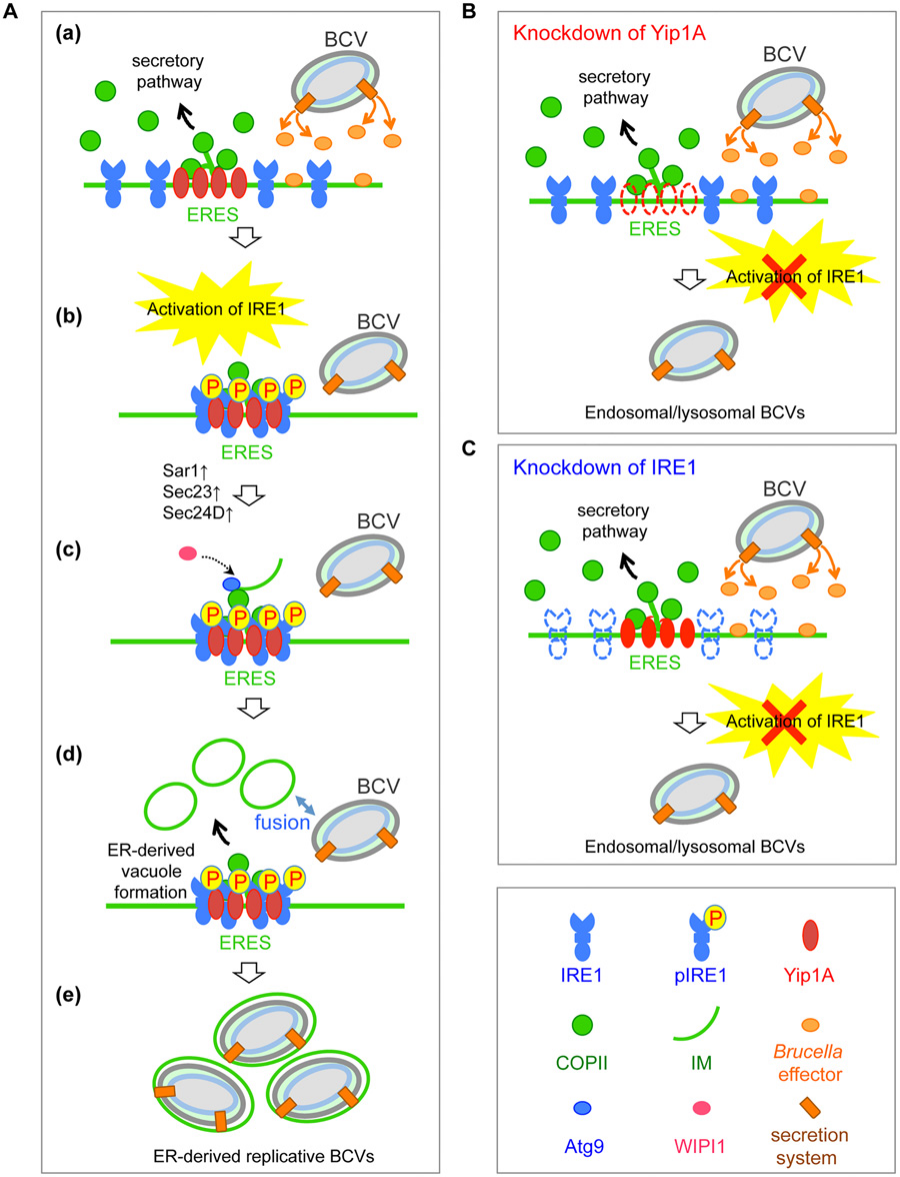
Proposed model of how *B*. *abortus* matures into ER-derived replicative BCVs. (A) (a) During infection, *B*. *abortus* triggers the activation of IRE1, presumably by secreting effector molecules into the cytoplasm of host cells through a secretion system. (b) IRE1 molecules form high-order complexes at ERES with the aid of Yip1A, which are activated by autophosphorylation. (c) The activated IRE1 in turn triggers the biogenesis of ER-derived vacuoles. Atg9 and WIPI1 may be recruited to the IM at ERES, and support the formation of ER-derived vacuoles. The upregulation of the COPII components Sar1, Sec23 and Sec24 could facilitate the formation of such vacuoles. (d) The ER-derived vacuoles then fuse with endolysosomal vesicles. Given that *B*. *abortus* that have reached the ER are located in late endosomal/lysosomal compartments, they should be able to fuse with these vacuoles. (e) Once they have acquired the ER-derived membrane, BCVs retain functional features of the ER, and multiplication of *B*. *abortus* in individual vacuoles might be supported through continual accretion of ER membranes derived from the IRE1-specific UPR. (B) Knockdown of Yip1A prevents the activation of IRE1. Consequently, ER-derived membranes are not generated for bacterial replication and *B*. *abortus* remains in endosomal/lysosomal compartments. (C) Knockdown of IRE1 leads to loss of the activation of IRE1. Consequently, ER-derived membranes are not generated for bacterial replication and *B*. *abortus* remains in endosomal/lysosomal compartments.

On the basis of our findings, we propose a model for the maturation of *B*. *abortus* into ER-derived replicative BCVs (Fig. 9A). During infection, *B*. *abortus* triggers the activation of IRE1, presumably by secreting effector molecules into the cytoplasm of host cells through a secretion system. IRE1 molecules form high-order complexes at ERES with the aid of Yip1A, which are activated by autophosphorylation. The activated IRE1 in turn triggers the biogenesis of ER-derived autophagic vacuoles. Atg9 and WIPI1 may be recruited to the IM at ERES, and support the formation of ER-derived vacuoles. The upregulation of the COPII components Sar1, Sec23 and Sec24 could facilitate the formation of such vacuoles. These vacuoles then fuse with endolysosomal vesicles. *B*. *abortus* might intercept this UPR-induced process of vacuole formation to acquire ER-derived membranes. Given that the bacteria that have reached the ER are located in late endosomal/lysosomal compartments [2], they would be able to fuse with these vacuoles. Once they have acquired the ER-derived membrane, BCVs retain functional features of the ER, and multiplication of *B*. *abortus* in individual vacuoles might be supported through continual accretion of ER membranes derived from the IRE1-specific UPR. In contrast, the knockdown of Yip1A (Fig. 9B) or IRE1 (Fig. 9C) prevents the activation of IRE1, and therefore ER-derived membranes are not available for *Brucella* replication. Consequently, BCVs remain in endosomal/lysosomal compartments.

The proposed model explains many previous findings. For example, functional ERES, but not the subsequent secretory pathway, are required for the biogenesis of replicative BCVs and Sar1 mediates the fusion event between BCVs and the ER at ERES. COPII complexes are formed in close proximity to BCVs [3]. The extensive *Brucella* replication is linked to an accretion of ER, which could originate from the ER membrane [3]. *Brucella* exploits the host autophagic machinery to reach its replication compartment [5, 6]. These earlier reports did not address the mechanism by which the interaction of BCVs with Sar1/ERES and COPII complexes enables the bacteria to mature into ER-derived replicative BCVs. In the present study, we characterized the interplay between the host and pathogen at the molecular level, thereby showing how *B*. *abortus* subverts the host UPR and ER-derived vacuole formation machineries to mature into ER-derived replicative BCVs.

Here, we identified a novel role for Yip1A in the biogenesis of ER-derived replicative BCVs through activation of the IRE1 pathway of the UPR. To date, several functions in membrane trafficking have been suggested for Yip1A, including involvement in COPII vesicle budding at ERES [24], vesicle tethering to the Golgi membrane [37], and COPI-independent retrograde vesicle transport [38]. Recently, Dykstra et al. [39] reported that ER morphology was affected by the depletion of Yip1A, but we did not observe such whorled ER formation in our present study, presumably because the event occurs after long-term treatment with Yip1A siRNA (48–72 hr) in contrast to the shorter-term treatment in our study (24 hr). In the present study, BCVs were confined within Lamp2-positive compartments by the depletion of Yip1A. This implies that Yip1A may play an additional role in trafficking from the endosomal/lysosomal compartments to the ER to generate ER-derived BCVs. Kano et al. [38] proposed that Yip1A regulates retrograde trafficking to the ER, which is associated with membrane recruitment of Rab6. Chen and Machner [40] demonstrated that *Legionella pneumophilla* (*L*. *pneumophilla*) secretes an effector protein LidA through its Dot/Icm type IV secretion system to recruit Rab6 on Legionella-containing vacuoles (LCVs), which is required for efficient intracellular replication of the pathogen. In the present study, however, knockdown of IRE1 yielded similar effects to those of Yip1A-knockdown: BCVs were locked in a Lamp2-positive stage devoid of ER-derived membrane, the upregulation of the COPII components was suppressed, and ER-derived vacuoles were diminished. These results presumably indicate that the transition from endosomal/lysosomal to ER-derived BCVs occurs not via trafficking to the ER but via a fusogenic event with ER-derived vacuoles, and that Yip1A-mediated activation of IRE1 at ERES is required for the formation of these vacuoles.

Infection with *B*. *abortus* upregulated the COPII vesicle components Sar1, Sec23 and Sec24D, which might enhance the capacity of COPII vesicles to export from ERES. Several recent studies suggest that ER-derived COPII vesicles are destined not only for the early secretory pathway to the Golgi, but also for autophagy [29, 32, 33], and that ERES play a key role in autophagosome formation [30, 31]. Newly budded COPII vesicles at ERES might function as a structural core and membrane source during the autophagosome formation [29, 32]. Tan et al. [33] indicate that COPII vesicles are diverted from the secretory pathway to the autophagic pathway, and supply membranes for autophagosome formation. *Brucella* spp. might modulate this intracellular trafficking via multiple effectors. Myeni et al. [19] demonstrated that the *Brucella* effectors BspB and BspF inhibited the host early secretory pathway prior to the biogenesis of replicative BCVs. These effectors might function to interrupt the secretory pathway in host cells and to redirect vesicle trafficking to other membrane compartments. Given the dual function of Yip1A in COPII vesicle formation or budding at ERES [24] and in regulating the activation of IRE1 pathway of the UPR (this study), Yip1A might coordinate the intersection between the secretory and ER-derived autophagic pathways at ERES.

Our model is in line with previous studies that demonstrate an intriguing link between the UPR and autophagic vacuole formation [27, 28, 41, 42]. Ogata et al. [41] demonstrated that the IRE1 signaling pathway is required for the activation of autophagy under the UPR. They showed that the PERK and ATF6 pathways are not needed for the activation of autophagy. Our data also suggest that the IRE1 pathway can regulate autophagic events independently from the other ER sensors, and plays a crucial role in supporting *B*. *abortus* replication. This result is consistent with the findings of Qin et al. [13], which demonstrated that *Brucella* replication is suppressed following the knockdown of IRE1. They also showed that the PERK and ATF6 pathways are not required for *Brucella* replication. On the other hand, Smith et al. [15] reported that all three UPR pathways are activated by *Brucella* infection in macrophages, and the *Brucella* TcpB is required for induction of the UPR. This might reflect a difference between macrophages and epithelial cells. *Brucella* exploits autophagy to establish a replicative niche in epithelial cells [4, 5], but not in macrophages [43]. Alternatively, multiple bacterial effectors might contribute to the induction of the UPR.

In the present study, we found that the autophagy-related proteins Atg9 and WIPI1 (one of mammalian homologues of yeast Atg18), but not DFCP1, are required for the biogenesis of the replicative BCVs. Atg9 is one of the most upstream proteins in the autophagy pathway and is targeted to autophagosome formation sites [36, 44, 45]. WIPI1 and DFCP1 translocate to the IM [34, 35], but localize to a spatially distinct structure [35]. In yeast, both Atg9 and Atg18 co-localize at the edge of the IM and are associated with ERES [44]. Physical interaction between Atg9 or Atg18 and the COPII components Sec23 [29] may support the recruitment of these proteins to ERES and anchor the IM to the ER. Given these findings, the COPII-dependent, ER-derived autophagic vesicle formation at ERES may be triggered by the Yip1A-mediated activation of IRE1, and provide membrane for the generation of replicative BCVs. Unlike canonical autophagy, the process requires Atg9 and WIPI1, but not DFCP1. Starr et al. [46] showed that depletion of the autophagy-initiation proteins ULK1 and Beclin1 and the autophagy-elongation proteins Atg5, Atg7, Atg16L1, and LC3B did not affect the maturation of endosomal BCVs into replicative BCVs. They also found that the autophagy-initiation proteins but not the autophagy-elongation proteins are required for the conversion of BCVs into a compartment with autophagic features at a later stage of infection (48–72 hr p.i.). In our study, we revealed that a non-canonical autophagic process at ERES, which involves Atg9 and WIPI1, but not DFCP1, is required for the biogenesis of the ER-derived membrane compartments during intracellular replication of *B*. *abortus*. Taken these findings together, we assume that selective autophagy-associated machineries of host cells may be utilized by the bacteria depending on the stage of their intracellular life.

Smith et al. [15] suggest that the IRE1-JNK signaling pathway, rather than the IRE1-XBP1 pathway, supports *Brucella* replication in macrophages. In the present study, the levels of spliced-XBP1 were not reduced as drastically as IRE1 phosphorylation upon knockdown of Yip1A. This might indicate that other downstream pathways of IRE1 are also affected by the knockdown of Yip1A. In addition to the splicing of XBP1 mRNA, activated IRE1 also transmits signals through the TNFR-associated factor-2 (TRAF2) and c-Jun NH_2_-terminal kinase (JNK) pathway. It also modulates the NF-κB and extracellular signal-regulated kinase signaling pathways. Bernales et al. [27] showed that expression of Hac1 (the yeast XBP1 homolog) was insufficient to induce autophagosome formation, which indicates that other signaling pathways besides XBP1 are required. Ogata et al. [41] suggest that activation of autophagy during the UPR is mediated by the IRE1-TRAF2-JNK pathway. Thus, signaling through the IRE1-JNK pathway might be important for *Brucella* replication.

In summary, we provide the first evidence that the novel host factor Yip1A plays a pivotal role in intracellular *Brucella* replication through the activation of the IRE1 pathway and the subsequent formation of ER-derived vacuoles. Characterization of the functions of the host factor Yip1A will provide new insights into the molecular mechanisms by which *Brucella* spp. replicates.

## Materials and Methods

### Bacterial strains

*Brucella abortus* strain 544 was obtained from the National Institute of Animal Health, Ibaraki, Japan and cultured on trypticase soy agar with 5% sheep blood (Nippon Becton Dickinson) at 37°C in a 10% CO_2_ atmosphere.

### Cell culture

HeLa cells (already-existing collection in Murata Laboratory at the University of Tokyo) were cultured at 37°C in a 5% CO_2_ atmosphere in Dulbecco’s Modified Eagle’s Medium (DMEM; Nissui) supplemented with 10% fetal calf serum (FCS) and penicillin/streptomycin (Gibco). For infection, HeLa cells were inoculated into DMEM supplemented with 10% FCS (DMEM-10%FCS) in 6-well tissue culture plates 24 hr before infection. For transfection, cells were seeded in 35-mm culture dishes. For confocal microscopy, cells were plated onto coverslips in 35-mm culture dishes. To induce the UPR, HeLa cells were treated with 5 μg/ml tunicamycin (Sigma) in DMEM and incubated at 37°C in a 5% CO_2_ atmosphere.

### Antibodies

The primary antibodies used were: mouse monoclonal anti-ERGIC53 (Alexis), rabbit monoclonal anti-GM130 (Abcam), rabbit polyclonal anti-Rab1 (Santa Cruz), mouse monoclonal anti-Rab2 (Abcam), rabbit polyclonal anti-Sec23 (Abcam), rabbit polyclonal anti-Sec24A (Proteintech), rabbit polyclonal anti-Sec24B (Sigma), rabbit polyclonal anti-Sec24C (Sigma), rabbit polyclonal anti-Sec24D (Sigma), mouse monoclonal anti-Sec31A (BD Biosciences), goat polyclonal anti-Sec61α (Abcam), mouse monoclonal anti-HSP47 (Enzo Life Sciences), rat monoclonal anti-HA (Roche), mouse monoclonal anti-GAPDH (Millipore), rabbit polyclonal anti-IRE1 (phospho S724; Abcam), rabbit polyclonal anti-IRE1 (Abcam), rabbit monoclonal anti-phospho-PERK (Thr980; Cell Signaling Technology), rabbit polyclonal anti-ATF6 (Abcam), rabbit polyclonal anti-XBP1 (Abcam), rabbit polyclonal anti-Sar1 (Abcam), mouse monoclonal anti-Lamp2 (developed by J. T. August and J. E. K. Hildreth, obtained from the Developmental Studies Hybridoma Bank, created by the NICHD of the NIH, and maintained at The University of Iowa), mouse monoclonal anti-β-tubulin (Sigma), rabbit monoclonal anti-Atg9A (Cell Signaling Technology), rabbit polyclonal anti-WIPI1 (Cell Signaling Technology), rabbit polyclonal anti-ZFYVE1/DFCP1 (Abcam), and mouse monoclonal anti-calnexin (BD Biosciences) antibodies. The rabbit polyclonal anti-Yip1A antibody was raised as described in Kano et al. [38]. The guinea pig polyclonal anti-Yip1A antibody was generated by MBL (Medical and Biological Laboratories) against the Yip1A peptide MMQPQQPYTGQIYQPTQC. The polyclonal anti-*Brucella abortus* antibody was purified from rabbit serum immunized with formalin-inactivated whole cells of *B*. *abortus* 544. The secondary antibodies used for immunofluorescence were: Alexa Fluor 488 Goat Anti-Rabbit IgG (H+L) (Life Technologies), Alexa Fluor 488 Goat Anti-Mouse IgG (H+L) (Life Technologies), Alexa Fluor 488 Goat Anti-rat IgG (H+L) (Life Technologies), Cy3-conjugated Goat Anti-Rabbit IgG (Chemicon), Cy3-conjugated Goat Anti-Mouse IgG (Chemicon), Alexa Fluor 647 Goat Anti-mouse IgG (H+L) (Life Technologies), and Alexa Fluor 647 Goat Anti-Guinea Pig IgG (H+L) (Life Technologies) antibodies. The secondary antibodies used for Western blotting were: Horse Radish Peroxidase (HRP)-conjugated Goat Anti-Mouse IgG (Promega), HRP-conjugated Anti-Goat IgG (Santa Cruz), and HRP-conjugated Goat Anti-Rabbit IgG (Cell Signaling) antibodies. Normal rabbit IgG was purchased from Santa Cruz.

### siRNA and transfection

Small interfering RNA (siRNA) against human Yip1A (ID 127564), siRNA against human IRE1 (s200430) and negative control siRNA (Silencer Negative Control 1 siRNA) were obtained from Ambion, and siRNAs against human Atg9 (SASI_Hs02_00312185), WIPI1 (SASI_Hs01_00100302) and DFCP1/ZFYVE (SASI_Hs01_00116581) were purchased from Sigma. FuGENE HD Transfection Reagent (Roche) was used for plasmid transfection, and Lipofectamine 2000 Transfection Reagent (Invitrogen) was used for siRNA transfection. siRNA was transfected 1 h after *Brucella* infection.

### Infections

HeLa cells were infected with log-phase cultures of *B*. *abortus* at a multiplicity of infection (MOI) of 400. The culture plates were centrifuged at 1,000 × g for 10 min at 20°C and then incubated for 1 hr at 37°C in a 5% CO_2_ atmosphere. After washing twice with DMEM-10%FCS, the cells were incubated for 1 hr in DMEM-10%FCS supplemented with 50 μg/ml gentamicin to kill extracellular bacteria. Thereafter, the culture medium was replaced by DMEM-10%FCS supplemented with 10 μg/ml gentamicin.

### Determination of CFUs

To evaluate intracellular *Brucella* growth, infected cells were washed three times with PBS and lysed with 0.5 ml of 0.1% Triton X-100 in PBS. Serial dilutions of the lysates were plated onto Thayer-Martin Agar (Nippon Becton Dickinson) and incubated for 3 days at 37°C in a 5% CO_2_ atmosphere before CFUs were counted.

### Immunofluorescence microscopy

HeLa cells were washed twice with PBS, fixed and permeabilized with methanol-acetone (1:1, v/v) for 6.5 min at 4°C, and then washed three times with PBS. The cells were blocked for 30 min in PBS that contained 3% bovine serum albumin (BSA) and incubated with the respective primary antibody in blocking buffer for 2 hr at room temperature. After washing three times with PBS, the cells were incubated with the respective secondary antibody in blocking buffer for 1 hr at room temperature. After washing three times with PBS, the coverslips were mounted in SlowFade Gold antifade reagent (Invitrogen) and examined under oil immersion on a Zeiss LSM 510 laser scanning confocal microscope.

### Electron microscopy

The ultrastructure of HeLa cells infected with *B*. *abortus* was examined by transmission electron microscopy. Infected HeLa cells were prefixed with 2.5% glutaraldehyde and 2% paraformaldehyde in 0.1 M phosphate buffer, pH 7.4 for 2 hr at room temperature, postfixed in 1% osmium tetroxide, and embedded in Epon. Ultrathin sections were stained with uranyl acetate and lead citrate, and then observed under a transmission electron microscope (H-7650, Hitachi Ltd.) at 80 kV.

### Immunoprecipitation

HeLa cells were scraped into ice-cold lysis buffer (50 mM Tris, pH 8.0, 150 mM NaCl, 1% NP 40, 0.1% SDS, 0.5% sodium deoxycholate) that contained protease inhibitor cocktail and passed 15 times through a 27-gauge needle. The cells were incubated for 30 min at 4°C with rotation and centrifuged for 20 min at 15,000 rpm. The supernatant was immunoprecipitated with rabbit anti-IRE1 (phospho S724) antibody or normal rabbit IgG for 3 hr at 4°C followed by Protein G Sepharose 4 Fast Flow (GE Healthcare) overnight at 4°C. After centrifugation at 13,000 rpm for 5 s, the precipitates were washed three times with lysis buffer and then boiled in 2× SDS sample buffer for 5 min. The immunoprecipitates were separated by SDS-PAGE and immunoblotted with rabbit anti-Yip1A antibody.

### SDS-PAGE and Western blotting

HeLa cells were scraped into RIPA buffer (50 mM Tris, pH 7.4, 150 mM NaCl, 1% NP 40, 0.25% sodium deoxycholate, 1 mM EDTA) that contained protease inhibitor cocktail (Roche) and passed 30 times through a 27-gauge needle. The cell lysates were mixed with 2× SDS sample buffer and boiled for 5 min. Proteins were separated on a 5–20% SDS polyacrylamide gel, and transferred onto PVDF membrane (Millipore). The membrane was blocked for 1 hr at room temperature with TBS that contained 0.1% Tween 20 (TBST) and 5% BSA, and then incubated with the respective primary antibody in blocking buffer overnight at 4°C. After washing three times with TBST, the membrane was incubated with the respective secondary antibody in blocking buffer for 1 hr at room temperature. After washing three times with TBST, protein bands were detected using the ECL Western Blotting Detection Kit (Amersham) and a LAS-4000 mini imaging system (FUJIFILM). The intensity of the bands was quantified using the MultiGauge software (FUJIFILM).

### Native PAGE

HeLa cells were scraped into 50mM Tris-buffered saline (TBS) that contained 1% Triton X-100 and protease inhibitor cocktail, and passed 30 times through a 27-gauge needle. The cell lysates were mixed with 2× Native PAGE loading buffer (Cosmo Bio). The same amounts of protein were loaded in each lane of a 5%~20% native gel. The electrophoresis ran at 10mA for 2.5hr at 4°C, and then the gel was subjected to Western blot analysis with a pIRE1 antibody.

### RNA isolation and RT-PCR

Total RNA was purified from *Brucella*-infected HeLa cells using an RNeasy Mini Kit (Qiagen) and reverse-transcribed with the use of a ReverTra Ace qPCR RT Kit (TOYOBO Co. Ltd.). One-step PCR was carried out using Fast SYBR Green Master Mix (Applied Biosystems) and a StepOnePlus Real-Time PCR System (Applied Biosystems). The primer pairs used were: forward, 5’-GCGAATTCTCATCCAGTTTGGCTATGTA-3’ and reverse 5’-GCGTCGACTCACTGTCCTTCCATGGCTAA-3’ for Yip1A, forward, 5’-GGTCTGCTGAGTCCGCAGCAGG-3’ and reverse, 5’-GGGCTTGGTATATATGTGG-3’ for spliced-XBP1, and forward, 5’-GCCATCAATGACCCCTTCATTGACC-3’ and reverse, 5’-CGCCTGCTTCACCACCTTCTTGATG-3’ for GAPDH. GAPDH was used as an internal standard.

### Statistical analysis

Differences between individual sets of data were assessed using a Welch’s t-test. Differences were considered significant at p < 0.05.

## Supporting Information

S1 Fig Replication of *B*. *abortus* within HeLa cells. (A) Intracellular growth of *B*. *abortus* within HeLa cells. HeLa cells were infected with *B*. *abortus* and CFUs were determined at 1, 12, and 24 hr p.i. Data are means ± SD from three independent experiments. (B) Representative confocal micrograph of HeLa cells infected with *B*. *abortu*s at 24 hr p.i. Fixed cells were stained for *B*. *abortus* (green). The infected cell is outlined with white dashed lines. Scale bar is 10 μm.
(TIF)

S2 Fig The interaction of Yip1A with pIRE1 is enhanced upon Tm treatment. Representative immunoblot showing the co-immunoprecipitation of Yip1A with pIRE1. After 0 hr or 5 hr of Tm treatment, immunoprecipitation was performed on HeLa cell lysates with an anti-pIRE1 antibody (lane labeled ‘pIRE1’) or control anti-rabbit IgG (lane labeled ‘IgG’), and the immunoprecipitates were analyzed by Western blotting with an anti-Yip1A antibody. The intensity of the bands was quantified using the MultiGauge software, and the results are shown in the bar graph. The protein levels at 0 hr of Tm treatment were assigned the value 1. Data are means ± SD from three independent experiments. *: p<0.05.
(TIF)

S3 Fig Depletion of Yip1A with siRNA and Western blot analysis of the UPR during Tm treatment. HeLa cells were transfected with each siRNA for 24 hr, and then treated with Tm to induce the UPR. Cell lysates were prepared at the indicated time points and analyzed by Western blotting. (A) Representative immunoblots showing the efficiency of Yip1A knockdown in HeLa cells at 24 hr after siRNA transfection. GAPDH was used for normalization. The intensity of the bands was quantified using the MultiGauge software, and the results are shown in the bar graph. The protein levels in control cells were assigned the value 1. Data are means ± SD from three independent experiments. **: p<0.01. (B) Representative confocal micrographs of control (left-hand panel) and Yip1A-knockdown (right-hand panel) cells stained for Yip1A, showing the depletion of Yip1A at 24 hr after siRNA transfection. Cells are outlined with white dashed lines. Scale bars are 10 μm. (C) Representative immunoblots for IRE1 and GAPDH, and relative protein levels of IRE1 in control (solid circles) and Yip1A-knockdown (open circles) cells during Tm treatment. GAPDH was used for normalization. The intensity of the bands was quantified using the MultiGauge software, and the results are shown in the line graph. The protein levels in control cells at the beginning of the Tm treatment were assigned the value 1. Data are means ± SD from three independent experiments. (D) Representative immunoblots for pPERK, cleaved-ATF6 and GAPDH, and relative protein levels of pPERK and cleaved-ATF6 in control (solid circles) and Yip1A-knockdown (open circles) cells during Tm treatment, showing the activation of PERK and ATF6. GAPDH was used for normalization. The intensity of the bands was quantified using the MultiGauge software, and the results are shown in the line graphs. The protein levels in control cells at the beginning of the Tm treatment were assigned the value 1. Data are means ± SD from three independent experiments. (E) Representative immunoblot showing the efficiency of IRE1 knockdown in HeLa cells at 24 hr after siRNA transfection. GAPDH was used for normalization. The intensity of the bands was quantified using the MultiGauge software, and the results are shown in the bar graph. The protein levels in control cells were assigned the value 1. Data are means ± SD from three independent experiments. **: p<0.01. (F) Representative confocal micrographs showing the localization of total IRE1 in control (left-hand panels) and Yip1A-knockdown (right-hand panel) cells. HeLa cells were transfected with each siRNA for 24 hr, and then treated with Tm for 5 hr to induce the UPR. Fixed cells were stained for IRE1. A magnification of the boxed area is shown below the main image. Several large vacuoles were observed in control cells (arrows), but not in Yip1A-knockdown cells. Scale bars are 10 μm.
(TIF)

S4 Fig Depletion of Atg9, WIPI1, and DFCP1 with siRNA. HeLa cells were transfected with each siRNA for 24 hr, and cell lysates were prepared and analyzed by Western blotting. (A-C) Representative immunoblots showing the knockdown efficiency of Atg9 (A), WIPI1 (B), and DFCP1 (C) in HeLa cells at 24 hr after siRNA transfection. GAPDH was used for normalization. The intensity of the bands was quantified using the MultiGauge software, and the results are shown in the bar graphs. The protein levels in control cells were assigned the value 1. Data are means ± SD from three independent experiments. **: p<0.01.
(TIF)

S5 Fig Depletion of Yip1A or IRE1 with siRNA during infection with *B*. *abortus*. HeLa cells were infected with *B*. *abortus*, and then transfected with each siRNA at 1 hr p.i. (A) Relative mRNA levels of Yip1A in control (solid circles) and Yip1A-knockdown (open circles) cells during infection with *B*. *abortus*. Total RNA was extracted at the indicated time points and RT-PCR was carried out as described in Materials and Methods. The mRNA levels at time 0 hr were assigned the value 1. Data are means ± SD from three independent experiments. (B) Representative immunoblots for Yip1A and GAPDH, and relative protein levels of Yip1A in control (solid circles) and Yip1A-knockdown (open circles) cells during infection with *B*. *abortus*. Cell lysates were collected at the indicated time points, and analyzed by Western blotting. GAPDH was used for normalization. The intensity of the bands was quantified using the MultiGauge software, and the results are shown in the line graph. The protein levels at time 0 hr were assigned the value 1. Data are means ± SD from three independent experiments. (C) Representative immunoblots for IRE1 and GAPDH, and relative protein levels of IRE1 in control (solid circles) and Yip1A-knockdown (open circles) cells during *Brucella* infection. Cell lysates were collected at the indicated time points, and analyzed by Western blotting. GAPDH was used for normalization. The intensity of the bands was quantified using the MultiGauge software, and the results are shown in the line graph. The protein levels at time 0 hr were assigned the value 1. Data are means ± SD from three independent experiments. (D) Representative immunoblots for Sar1, Sec23, Sec24D, and GAPDH, and relative protein levels of Sar1, Sec23, and Sec24D in control uninfected (solid bars) and Yip1A-knockdown (open bars) cells at 24 hr p.i. Cell lysates were analyzed by Western blotting. GAPDH was used for normalization. The intensity of the bands was quantified using the MultiGauge software, and the results are shown in the bar graphs. The protein levels in control cells were assigned the value 1. Data are means ± SD from three independent experiments. *: p<0.05. (E) Representative immunoblots for IRE1 and GAPDH, and relative protein levels of IRE1 in control (solid circles) and IRE1-knockdown (open circles) cells during infection with *B*. *abortus*. Cell lysates were collected at the indicated time points, and analyzed by Western blotting. GAPDH was used for normalization. The intensity of the bands was quantified using the MultiGauge software, and the results are shown in the line graph. The protein levels at time 0 hr were assigned the value 1. Data are means ± SD from three independent experiments.
(TIF)

S6 Fig Depletion of Atg9, WIPI1, and DFCP1 with siRNA during infection with *B*. *abortus*. HeLa cells were infected with *B*. *abortus*, and then transfected with each siRNA at 1 hr p.i. Cell lysates were collected at the indicated time points, and analyzed by Western blotting. (A-C) Representative immunoblots and relative protein levels of Atg9 (A), WIPI1 (B), and DFCP1 (C) in control (solid circles) and respective knockdown (open circles) cells during infection with *B*. *abortus*. GAPDH was used for normalization. The intensity of the bands was quantified using the MultiGauge software, and the results are shown in the line graphs. The protein levels at time 0 hr were assigned the value 1. Data are means ± SD from three independent experiments.
(TIF)

## References

[1] Pappas G, Akritidis N, Bosilkovski M, Tsianos E. Brucellosis. N Engl J Med. 2005;352: 2325–2336. 15930423 10.1056/NEJMra050570

[2] Starr T, Ng TW, Wehrly TD, Knodler LA, Celli J. *Brucella* intracellular replication requires trafficking through the late endosomal/lysosomal compartment. Traffic 2008;9: 678–694. 10.1111/j.1600-0854.2008.00718.x 18266913

[3] Celli J, Salcedo SP, Gorvel JP. *Brucella* coopts the small GTPase Sar1 for intracellular replication. Proc Natl Acad Sci USA. 2005;102: 1673–1678. 15632218 10.1073/pnas.0406873102 PMC547823

[4] Pizarro-Cerda J, Meresse S, Parton RG, van der Goot G, Sola-Landa A, Lopez-Goñi I, et al *Brucella abortus* transits through the autophagic pathway and replicates in the endoplasmic reticulum of nonprofessional phagocytes. Infect Immun. 1998;66: 5711–5724. 9826346 10.1128/iai.66.12.5711-5724.1998 PMC108722

[5] Pizarro-Cerdá J, Moreno E, Sanguedolce V, Mége J-L, Gorvel JP. Virulent *Brucella abortus* avoids lysosome fusion and distributes within autophagosome-like compartments. Infect Immun. 1998;66: 2387–2392. 9573138 10.1128/iai.66.5.2387-2392.1998 PMC108212

[6] Celli J, de Chastellier C, Franchini DM, Pizarro-Cerda J, Moreno E, Gorvel JP. *Brucella* evades macrophage killing via VirB-dependent sustained interactions with the endoplasmic reticulum. J Exp Med. 2003;198: 545–556. 12925673 10.1084/jem.20030088 PMC2194179

[7] Hassan IH, Zhang MS, Powers LS, Shao JQ, Baltrusaitis J, Rutkowski DT, et al Influenza A viral replication is blocked by inhibition of the inositol-requiring enzyme 1 (IRE1) stress pathway. J Biol Chem. 2012;287: 4679–4689. 10.1074/jbc.M111.284695 22194594 PMC3281634

[8] Tardif KD, Mori K, Kaufman RJ, Siddiqui A. Hepatitis C virus suppresses the IRE1-XBP1 pathway of the unfolded protein response. J Biol Chem. 2004;279: 17158–17164. 14960590 10.1074/jbc.M312144200

[9] Su HL, Liao CL, Lin YL. Japanese encephalitis virus infection initiates endoplasmic reticulum stress and an unfolded protein response. J Virol. 2002;76: 4162–4171. 11932381 10.1128/JVI.76.9.4162-4171.2002 PMC155064

[10] Seimon TA, Kim MJ, Blumenthal A, Koo J, Ehrt S, Wainwright H. Induction of ER stress in macrophages of *tuberculosis* granulomas. PLoS One. 2010;5(9): e12772 10.1371/journal.pone.0012772 20856677 PMC2939897

[11] Baruch M, Belotserkovsky I, Hertzog BB, Ravins M, Dov E, Mclver KS, et al An extracellular bacterial pathogen modulates host metabolism to regulate its own sensing and proliferation. Cell. 2014;156: 97–108. 10.1016/j.cell.2013.12.007 24439371 PMC3926133

[12] Schroder M, Kaufman RJ. The mammalian unfolded protein response. Annu Rev Biochem. 2005;74: 739–789. 15952902 10.1146/annurev.biochem.73.011303.074134

[13] Qin QM, Pei J, Ancona V, Shaw BD, Ficht TA, de Figueiredo P. RNAi screen of endoplasmic reticulum-associated host factors reveals a role for IRE1alpha in supporting *Brucella* replication. PLoS Pathog. 2008;4: e1000110 10.1371/journal.ppat.1000110 18654626 PMC2453327

[14] de Jong MF, Starr T, Winter MG, den Hartigh AB, Child R, Knodler LA, et al Sensing of Bacterial Type IV Secretion via the Unfolded Protein Response. mBio. 2013;4: e00418–12. 10.1128/mBio.00418-12 23422410 PMC3624511

[15] Smith JA, Khan M, Magnani DD, Harms JS, Durward M, Radhakrishnan GK, et al *Brucella* induces an unfolded protein response via TcpB that supports intracellular replication in macrophages. PLoS Pathog. 2013;9: e1003785 10.1371/journal.ppat.1003785 24339776 PMC3855547

[16] de Jong MF, Sun YH, den Hartigh AB, van Dijl JM, Tsolis RM. Identification of VceA and VceC, two members of the VjbR regulon that are translocated into macrophages by the *Brucella* type IV secretion system. Mol Microbiol. 2008;70: 1378–1396. 10.1111/j.1365-2958.2008.06487.x 19019140 PMC2993879

[17] de Barsy M, Jamet A, Filopon D, Nicolas C, Laloux G, Rual JF, et al Identification of a *Brucella* spp. secreted effector specifically interacting with human small GTPase Rab2. Cell Microbiol. 2011;13: 1044–1058. 10.1111/j.1462-5822.2011.01601.x 21501366

[18] de Barsy M, Mirabella A, Letesson JJ, De Bolle X. A *Brucella abortus* cstA mutant is defective for association with endoplasmic reticulum exit sites and displays altered trafficking in HeLa cells. Microbiology. 2012;158: 2610–2618. 22820839 10.1099/mic.0.060509-0

[19] Myeni S, Child R, Ng TW, Kupko JJ 3rd, Wehrly TD, Porcella SF, et al *Brucella* modulates secretory trafficking via multiple type IV secretion effector proteins. PLoS Pathog. 2013;9: e1003556 10.1371/journal.ppat.1003556 23950720 PMC3738490

[20] Fugier E, Salcedo SP, de Chastellier C, Pophillat M, Muller A, Arce-Gorvel V, et al The glyceraldehyde-3-phosphate dehydrogenase and the small GTPase Rab 2 are crucial for *Brucella* replication. PLoS Pathog. 2009;5: e1000487 10.1371/journal.ppat.1000487 19557163 PMC2695806

[21] Yoshida H, Matsui T, Yamamoto A, Okada T, Mori K. XBP1 mRNA is induced by ATF6 and spliced by IRE1 in response to ER stress to produce a highly active transcription factor. Cell. 2001;107: 881–91. 11779464 10.1016/s0092-8674(01)00611-0

[22] Sriburi R, Bommiasamy H, Buldak GL, Robbins GR, Frank M, Jackowski S, et al Coordinate regulation of phospholipid biosynthesis and secretory pathway gene expression in XBP-1(S)-induced endoplasmic reticulum biogenesis. J Biol Chem. 2007;282: 7024–7034. 17213183 10.1074/jbc.M609490200

[23] D'Arcangelo JG, Stahmer KR, Miller EA. Vesicle-mediated export from the ER: COPII coat function and regulation. Biochim Biophys Acta. 2013;1833: 2464–2472. 10.1016/j.bbamcr.2013.02.003 23419775 PMC3676692

[24] Tang BL, Ong YS, Huang B, Wei S, Wong ET, Qi R, et al A membrane protein enriched in endoplasmic reticulum exit sites interacts with COPII. J Biol Chem. 2001;276: 40008–40017. 11489904 10.1074/jbc.M106189200

[25] Korennykh AV, Egea PF, Korostelev AA, Finer-Moore J, Zhang C, Shokat KM, et al The unfolded protein response signals through high-order assembly of Ire1. Nature. 2009;457: 687–693. 10.1038/nature07661 19079236 PMC2846394

[26] Li H, Korennykh AV, Behrman SL, Walter P. Mammalian endoplasmic reticulum stress sensor IRE1 signals by dynamic clustering. Proc Natl Acad Sci USA. 2010;107 (37): 16113–16118. 10.1073/pnas.1010580107 20798350 PMC2941319

[27] Bernales S, McDonald KL, Walter P. Autophagy counterbalances endoplasmic reticulum expansion during the unfolded protein response. PLoS Biology. 2006;4(12): e423 17132049 10.1371/journal.pbio.0040423 PMC1661684

[28] Hoyer-Hansen M, Jaattela M. Connecting endoplasmic reticulum stress to autophagy by unfolded protein response and calcium. Cell Death Differ. 2007;14: 1576–1582. 17612585 10.1038/sj.cdd.4402200

[29] Graef M, Friedman JR, Graham C, Babu M, Nunnari J. ER exit sites are physical and functional core autophagosome biogenesis components. Mol Biol Cell. 2013;24(18): 2918–2931. 10.1091/mbc.E13-07-0381 23904270 PMC3771953

[30] Zoppino FC, Militello RD, Slavin I, Alvarez C, Colombo MI. Autophagosome formation depends on the small GTPase Rab1 and functional ER exit sites. Traffic. 2010;11: 1246–1261. 10.1111/j.1600-0854.2010.01086.x 20545908

[31] Ge L, Melville D, Zhang M, Schekman R. The ER–Golgi intermediate compartment is a key membrane source for the LC3 lipidation step of autophagosome biogenesis. Elife. 2013;2: e00947 10.7554/eLife.00947 23930225 PMC3736544

[32] Wang J, Tan D, Cai Y, Reinisch KM, Walz T, Ferro-Novick S. A requirement for ER-derived COPII vesicles in phagophore initiation. Autophagy. 2014;10: 708–9. 10.4161/auto.28103 24561915 PMC4091162

[33] Tan D, Cai Y, Wang J, Zhang J, Menon S, Chou H-T, et al The EM structure of the TRAPPIII complex leads to the identification of a requirement for COPII vesicles on the macroautophagy pathway. Proc Natl Acad Sci USA. 2013;110: 19432–19437. 10.1073/pnas.1316356110 24218626 PMC3845172

[34] Axe EL, Walker SA, Manifava M, Chandra P, Roderick HL, Habermann A, et al Autophagosome formation from membrane compartments enriched in phosphatidylinositol 3-phosphate and dynamically connected to the endoplasmic reticulum. J Cell Biol. 2008;182:685–701. 10.1083/jcb.200803137 18725538 PMC2518708

[35] Itakura E, Mizushima N. Characterization of autophagosome formation site by a hierarchical analysis of mammalian Atg proteins. Autophagy. 2010;6: 764–776. 20639694 10.4161/auto.6.6.12709 PMC3321844

[36] Koyama-Honda I, Itakura E, Fujiwara TK, Mizushima N. Temporal analysis of recruitment of mammalian ATG proteins to the autophagosome formation site. Autophagy. 2013;9: 1491–9. 10.4161/auto.25529 23884233

[37] Jin C, Zhang Y, Zhu H, Ahmed K, Fu C, Yao X. Human Yip1A specifies the localization of Yif1 to the Golgi apparatus. Biochem Biophys Res Commun. 2005;334: 16–22. 15990086 10.1016/j.bbrc.2005.06.051

[38] Kano F, Yamauchi S, Yoshida Y, Watanabe-Takahashi M, Nishikawa K, Nakamura N, et al Yip1A regulates the COPI-independent retrograde transport from the Golgi complex to the ER. J Cell Sci. 2009;122: 2218–2227. 10.1242/jcs.043414 19509059

[39] Dykstra KM, Pokusa JE, Suhan J, Lee TH. Yip1A structures the mammalian endoplasmic reticulum. Mol Biol Cell. 2010;21: 1556–1568. 10.1091/mbc.E09-12-1002 20237155 PMC2861614

[40] Chen Y, Machner MP. Targeting of the small GTPase Rab6A’ by the *Legionella pneumophila* effector LidA. Infect Immun. 2013;81:2226–2235. 10.1128/IAI.00157-13 23569112 PMC3676037

[41] Ogata M, Hino S, Saito A, Morikawa K, Kondo S, Kanemoto S, et al Autophagy is activated for cell survival after endoplasmic reticulum stress. Mol Cell Biol. 2006;26(24): 9220–9231. 17030611 10.1128/MCB.01453-06 PMC1698520

[42] Li J, Ni M, Lee B, Barron E, Hinton DR, Lee AS. The unfolded protein response regulator GRP78/BiP is required for endoplasmic reticulum integrity and stress-induced autophagy in mammalian cells. Cell Death Differ. 2008;15: 1460–1471. 10.1038/cdd.2008.81 18551133 PMC2758056

[43] Arenas GN, Staskevich AS, Aballay A, Mayorga LS. Intracellular trafficking of *Brucella abortus* in J774 macrophages. Infect Immun. 2000;68: 4255–4263. 10858243 10.1128/iai.68.7.4255-4263.2000 PMC101738

[44] Suzuki K. Akioka M, Kondo-Kakuta C, Yamamoto H, Ohsumi Y. Fine mapping of autophagy-related proteins during autophagosome formation in *Saccharomyces cerevisiae* . J Cell Sci. 2013;126: 2534–44. 10.1242/jcs.122960 23549786

[45] Orsi A, Razi M, Dooley H, Robinson D, Weston AE, Collinson LM, et al Dynamic and transient interactions of Atg9 with autophagosomes, but not membrane integration, is required for autophagy. Mol Biol Cell. 2012;23(10): 1860–73. 10.1091/mbc.E11-09-0746 22456507 PMC3350551

[46] Starr T, Child R, Wehrly TD, Hansen B, Hwang S, Lopez-Otein C, et al Selective subversion of autophagy complexes facilitates completion of the *Brucella* intracellular cycle. Cell Host & Microbe. 2012;11: 33–45. 22264511 10.1016/j.chom.2011.12.002 PMC3266535

