# Supplementary figures and images for: Yip1A, a Novel Host Factor for the Activation of the IRE1 Pathway of the Unfolded Protein Response during Brucella Infection
Source: PLoS Pathog. 2015 Mar 5;11(3):e1004747. doi: 10.1371/journal.ppat.1004747 (PMC4350842; doi:10.1371/journal.ppat.1004747)

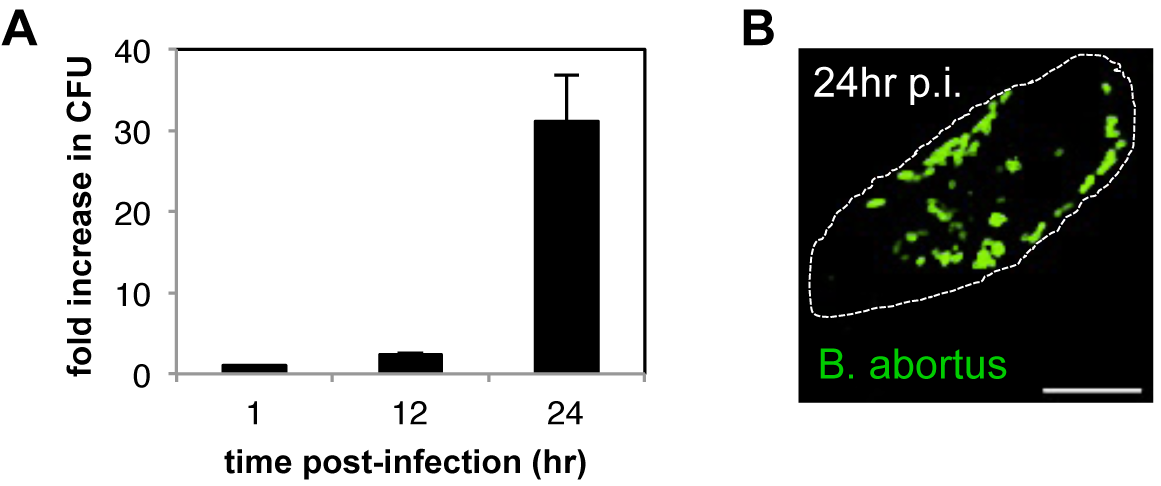

Supplement: S1 Fig — (A) Intracellular growth of B. abortus within HeLa cells. HeLa cells were infected with B. abortus and CFUs were determined at 1, 12, and 24 hr p.i. Data are means ± SD from three independent experiments. (B) Representative confocal micrograph of HeLa cells infected with B. abortus at 24 hr p.i. Fixed cells were stained for B. abortus (green). The infected cell is outlined with white dashed lines. Scale bar is 10 μm. (TIF) [file ppat.1004747.s001.tif]

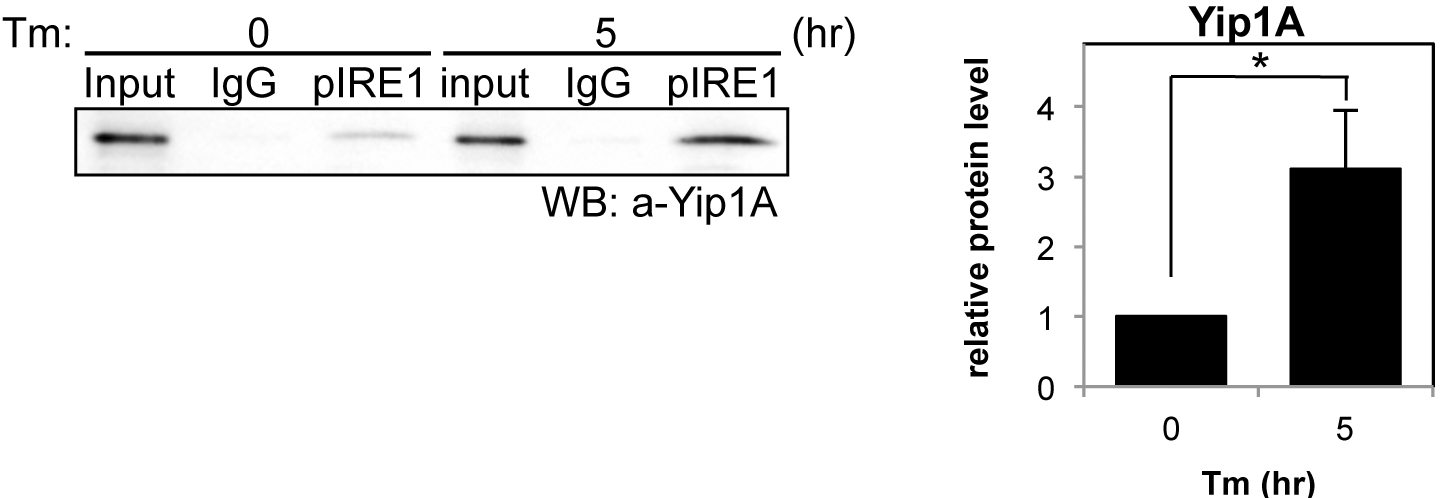

Supplement: S2 Fig — Representative immunoblot showing the co-immunoprecipitation of Yip1A with pIRE1. After 0 hr or 5 hr of Tm treatment, immunoprecipitation was performed on HeLa cell lysates with an anti-pIRE1 antibody (lane labeled ‘pIRE1’) or control anti-rabbit IgG (lane labeled ‘IgG’), and the immunoprecipitates were analyzed by Western blotting with an anti-Yip1A antibody. The intensity of the bands was quantified using the MultiGauge software, and the results are shown in the bar graph. The protein levels at 0 hr of Tm treatment were assigned the value 1. Data are means ± SD from three independent experiments. *: p<0.05. (TIF) [file ppat.1004747.s002.tif]

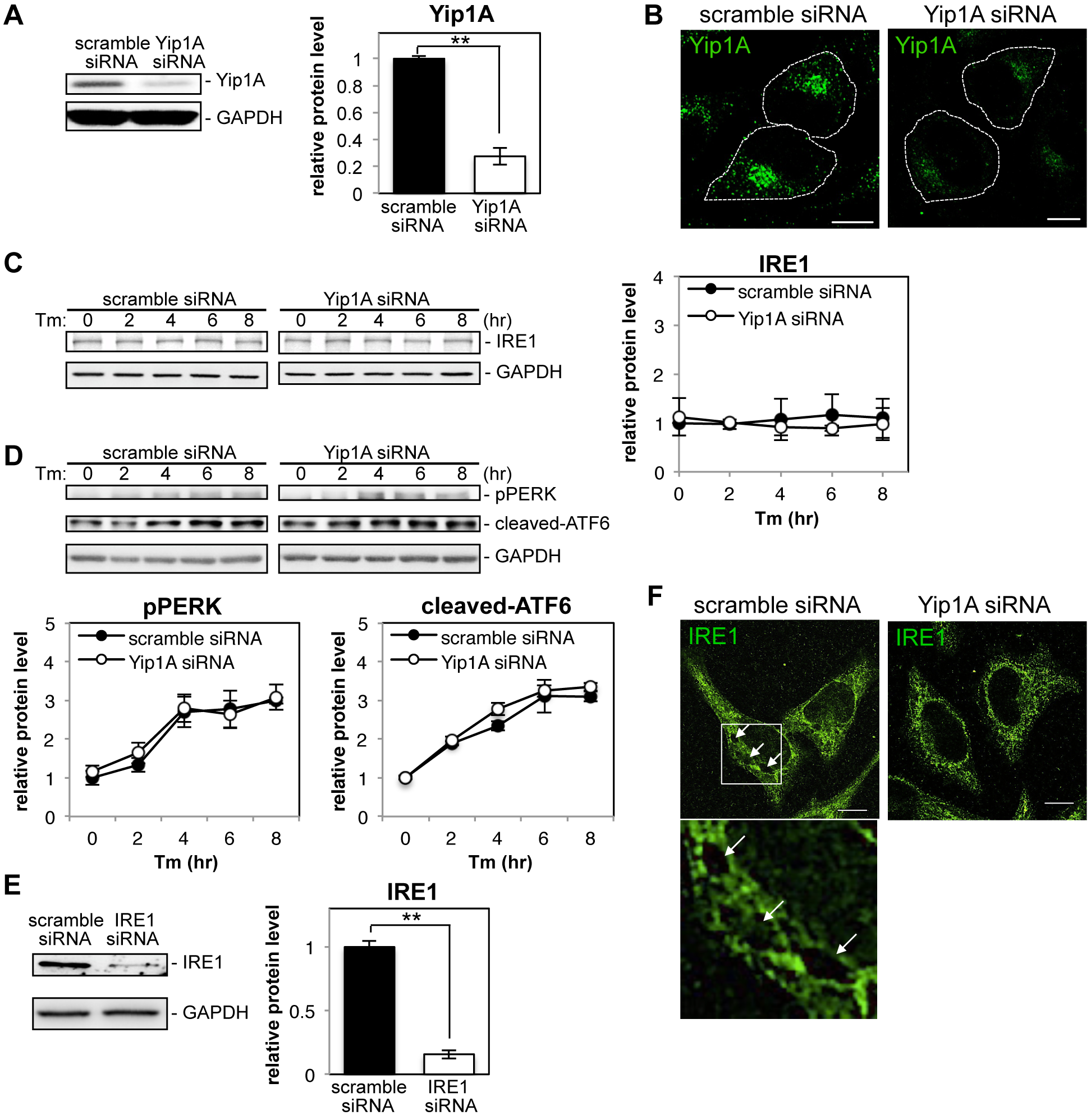

Supplement: S3 Fig — HeLa cells were transfected with each siRNA for 24 hr, and then treated with Tm to induce the UPR. Cell lysates were prepared at the indicated time points and analyzed by Western blotting. (A) Representative immunoblots showing the efficiency of Yip1A knockdown in HeLa cells at 24 hr after siRNA transfection. GAPDH was used for normalization. The intensity of the bands was quantified using the MultiGauge software, and the results are shown in the bar graph. The protein levels in control cells were assigned the value 1. Data are means ± SD from three independent experiments. **: p<0.01. (B) Representative confocal micrographs of control (left-hand panel) and Yip1A-knockdown (right-hand panel) cells stained for Yip1A, showing the depletion of Yip1A at 24 hr after siRNA transfection. Cells are outlined with white dashed lines. Scale bars are 10 μm. (C) Representative immunoblots for IRE1 and GAPDH, and relative protein levels of IRE1 in control (solid circles) and Yip1A-knockdown (open circles) cells during Tm treatment. GAPDH was used for normalization. The intensity of the bands was quantified using the MultiGauge software, and the results are shown in the line graph. The protein levels in control cells at the beginning of the Tm treatment were assigned the value 1. Data are means ± SD from three independent experiments. (D) Representative immunoblots for pPERK, cleaved-ATF6 and GAPDH, and relative protein levels of pPERK and cleaved-ATF6 in control (solid circles) and Yip1A-knockdown (open circles) cells during Tm treatment, showing the activation of PERK and ATF6. GAPDH was used for normalization. The intensity of the bands was quantified using the MultiGauge software, and the results are shown in the line graphs. The protein levels in control cells at the beginning of the Tm treatment were assigned the value 1. Data are means ± SD from three independent experiments. (E) Representative immunoblot showing the efficiency of IRE1 knockdown in HeLa cells at 24 hr after [file ppat.1004747.s003.tif]

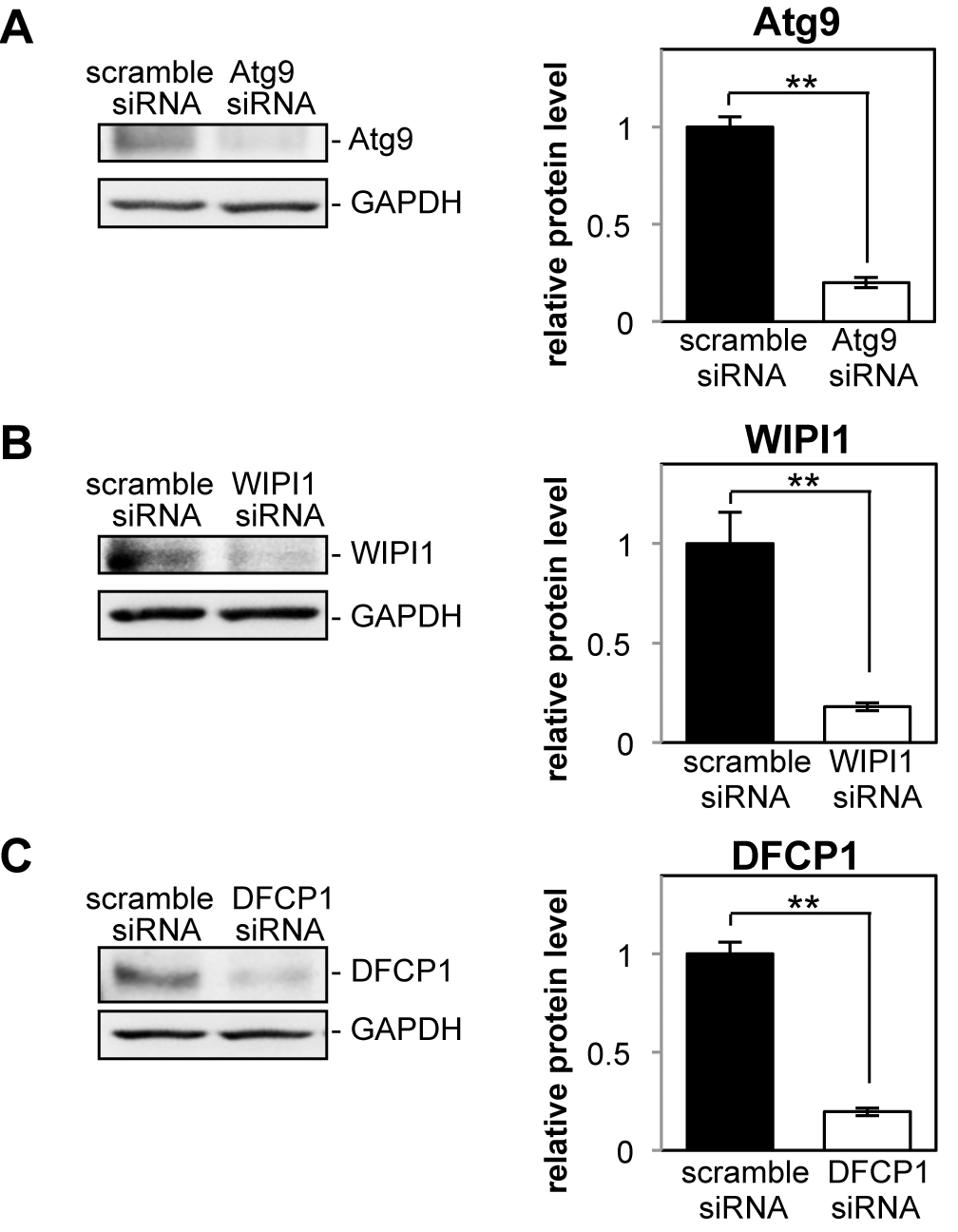

Supplement: S4 Fig — HeLa cells were transfected with each siRNA for 24 hr, and cell lysates were prepared and analyzed by Western blotting. (A-C) Representative immunoblots showing the knockdown efficiency of Atg9 (A), WIPI1 (B), and DFCP1 (C) in HeLa cells at 24 hr after siRNA transfection. GAPDH was used for normalization. The intensity of the bands was quantified using the MultiGauge software, and the results are shown in the bar graphs. The protein levels in control cells were assigned the value 1. Data are means ± SD from three independent experiments. **: p<0.01. (TIF) [file ppat.1004747.s004.tif]

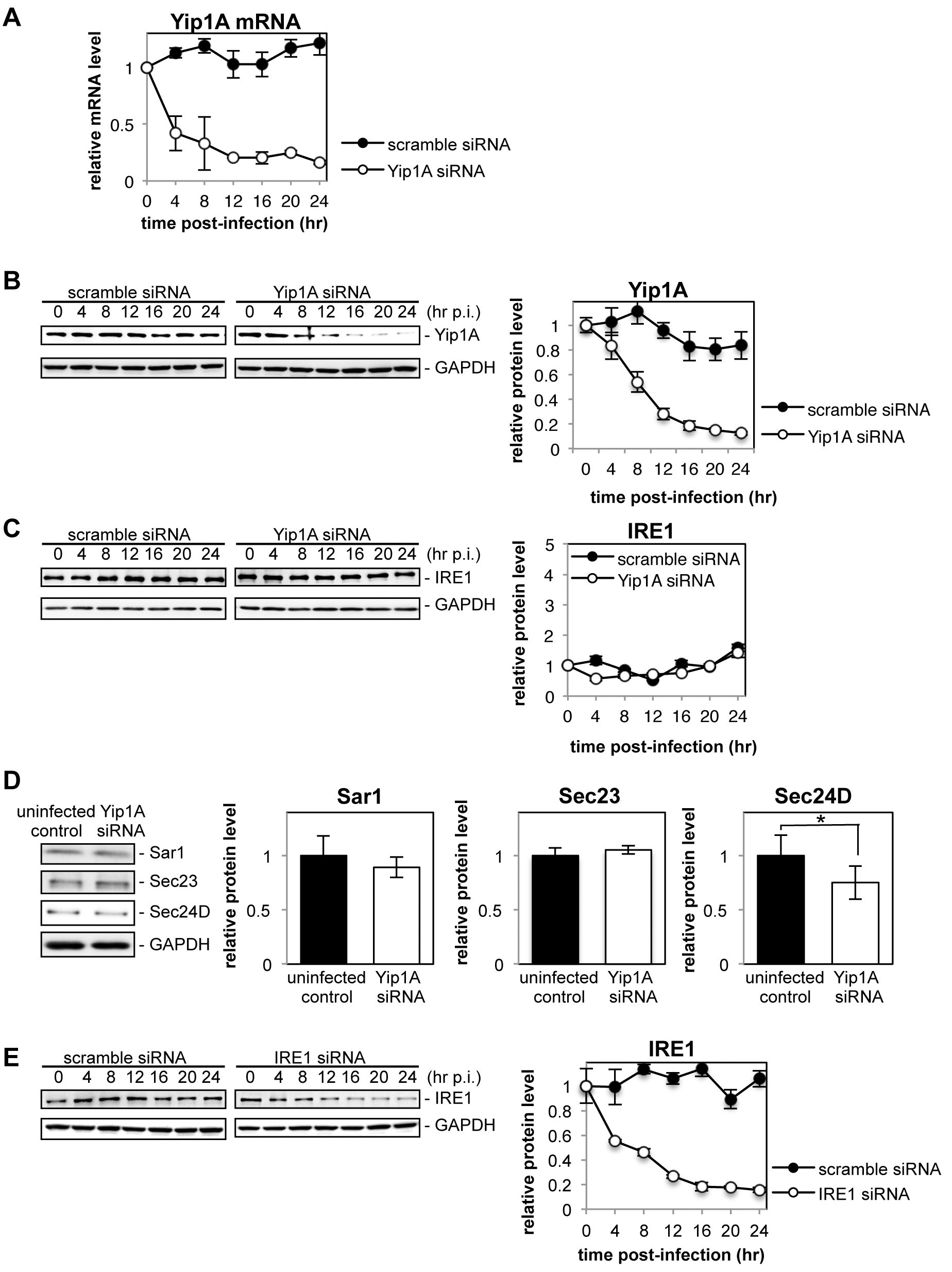

Supplement: S5 Fig — HeLa cells were infected with B. abortus, and then transfected with each siRNA at 1 hr p.i. (A) Relative mRNA levels of Yip1A in control (solid circles) and Yip1A-knockdown (open circles) cells during infection with B. abortus. Total RNA was extracted at the indicated time points and RT-PCR was carried out as described in Materials and Methods. The mRNA levels at time 0 hr were assigned the value 1. Data are means ± SD from three independent experiments. (B) Representative immunoblots for Yip1A and GAPDH, and relative protein levels of Yip1A in control (solid circles) and Yip1A-knockdown (open circles) cells during infection with B. abortus. Cell lysates were collected at the indicated time points, and analyzed by Western blotting. GAPDH was used for normalization. The intensity of the bands was quantified using the MultiGauge software, and the results are shown in the line graph. The protein levels at time 0 hr were assigned the value 1. Data are means ± SD from three independent experiments. (C) Representative immunoblots for IRE1 and GAPDH, and relative protein levels of IRE1 in control (solid circles) and Yip1A-knockdown (open circles) cells during Brucella infection. Cell lysates were collected at the indicated time points, and analyzed by Western blotting. GAPDH was used for normalization. The intensity of the bands was quantified using the MultiGauge software, and the results are shown in the line graph. The protein levels at time 0 hr were assigned the value 1. Data are means ± SD from three independent experiments. (D) Representative immunoblots for Sar1, Sec23, Sec24D, and GAPDH, and relative protein levels of Sar1, Sec23, and Sec24D in control uninfected (solid bars) and Yip1A-knockdown (open bars) cells at 24 hr p.i. Cell lysates were analyzed by Western blotting. GAPDH was used for normalization. The intensity of the bands was quantified using the MultiGauge software, and the results are shown in the bar graphs. The protein levels in control cells were [file ppat.1004747.s005.tif]

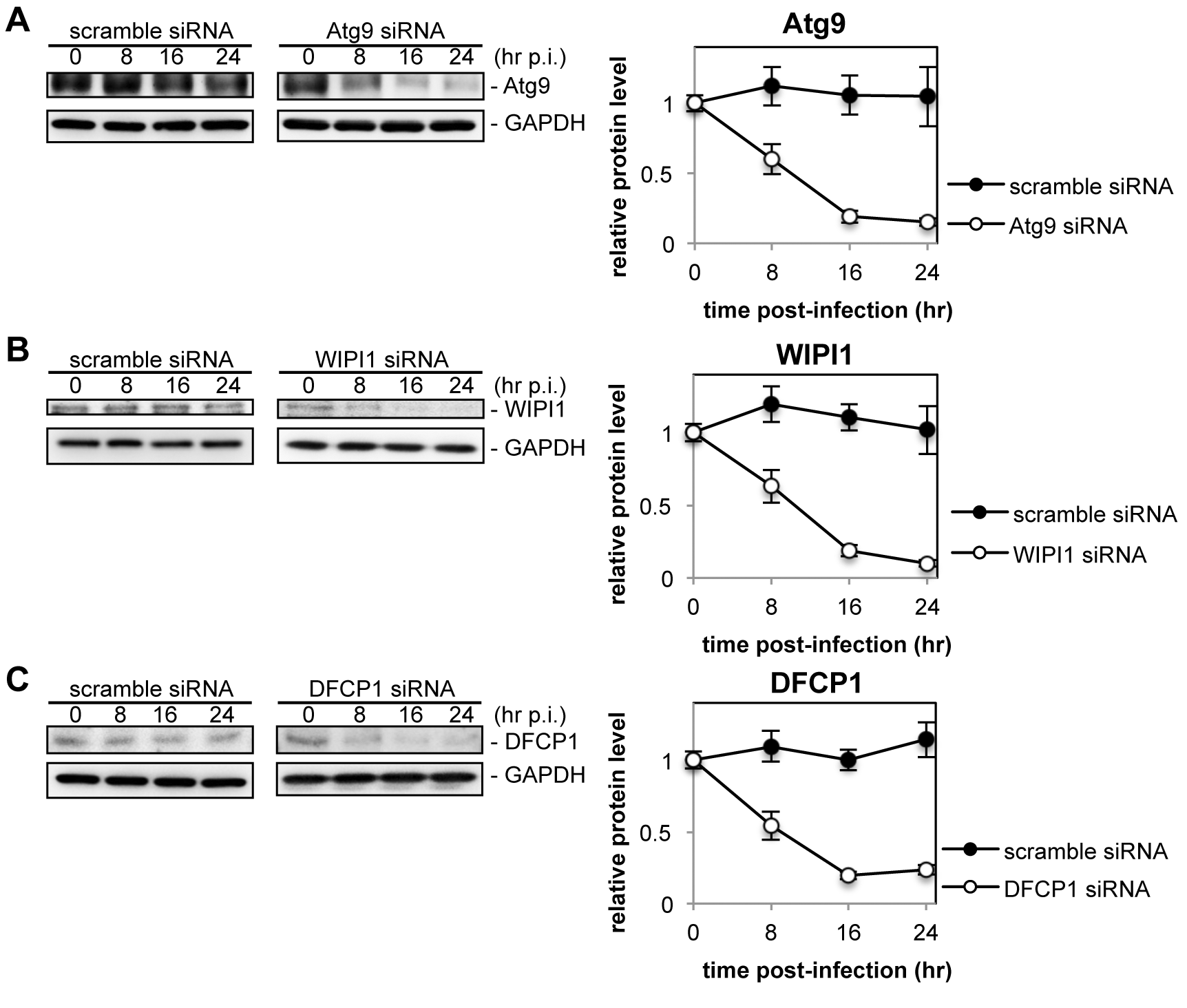

Supplement: S6 Fig — HeLa cells were infected with B. abortus, and then transfected with each siRNA at 1 hr p.i. Cell lysates were collected at the indicated time points, and analyzed by Western blotting. (A-C) Representative immunoblots and relative protein levels of Atg9 (A), WIPI1 (B), and DFCP1 (C) in control (solid circles) and respective knockdown (open circles) cells during infection with B. abortus. GAPDH was used for normalization. The intensity of the bands was quantified using the MultiGauge software, and the results are shown in the line graphs. The protein levels at time 0 hr were assigned the value 1. Data are means ± SD from three independent experiments. (TIF) [file ppat.1004747.s006.tif]
